# Molecular Interactions between Ionic Liquid Lubricants and Silica Surfaces: An MD Simulation Study

**DOI:** 10.1021/acs.jpcb.3c08397

**Published:** 2024-03-05

**Authors:** Mariana T. Donato, Rogério Colaço, Luis C. Branco, Benilde Saramago, José N. Canongia Lopes, Karina Shimizu, Adilson Alves de Freitas

**Affiliations:** † Centro de Química Estrutural, Institute of Molecular Sciences, Departamento de Engenharia Química, Instituto Superior Técnico, Universidade de Lisboa, Av. Rovisco Pais, 1049 001 Lisboa, Portugal; ‡ LAQV-REQUIMTE, Departamento de Química, NOVA School of Science and Technology, Universidade NOVA de Lisboa, Campus da Caparica, 2829-516 Caparica, Portugal; § IDMEC-Instituto de Engenharia Mecânica, Departamento de Engenharia Mecânica, Instituto Superior Técnico, Universidade de Lisboa, Av. Rovisco Pais, 1049-001 Lisboa, Portugal

## Abstract

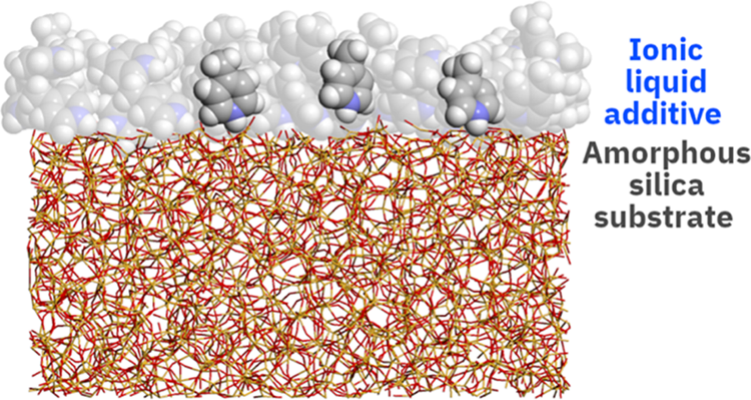

The unique physicochemical properties of ionic liquids (ILs) attracted interest in their application as lubricants of micro/nano-electromechanical systems. This work evaluates the feasibility of using the protic ionic liquids [4-picH][HSO_4_], [4-picH][CH_3_SO_3_], [MIMH][HSO_4_], and [MIMH][CH_3_SO_3_] and the aprotic ILs [C_6_mim][HSO_4_] and [C_6_mim][CH_3_SO_3_] as additives to model lubricant poly(ethylene glycol) (PEG200) to lubricate silicon surfaces. Additives based on the cation [4-picH]^+^ exhibited the best tribological performance, with the optimal value for 2% [4-picH][HSO_4_] in PEG200 (w/w). Molecular dynamics (MD) simulations of the first stages of adsorption of the ILs at the glass surface were performed to portray the molecular behavior of the ILs added to PEG200 and their interaction with the silica substrate. For the pure ILs at the solid substrates, the MD results indicated that weak specific interactions of the cation with the glass interface are lost to accommodate the larger anion in the first contact layer. For the PEG200 + 2% [4-picH][HSO_4_] system, the formation of a more compact protective film adsorbed at the glass surface is revealed by a larger *trans* population of the dihedral angle –O(R)–C–C–O(R)– in PEG200, in comparison to the same distribution for the pure model lubricant. Our findings suggest that the enhanced lubrication performance of PEG200 with [4-picH][HSO_4_] arises from synergistic interactions between the protic IL and PEG200 at the adsorbed layer.

## 1 Introduction

Molecular dynamics (MD) simulations have been considered a powerful tool to investigate the structure of ionic liquids (ILs) and their interactions with different types of surfaces, either as a complement to experiments or for predictive analysis. Several authors investigated the microscopic structures of 1-butyl-3-methylimidazolium hexafluorophosphate ([BMIM][PF_6_])^1,2^ and 1-octyl-3-methylimidazolium hexafluorophosphate ([OMIM][PF_6_])^2^ on hydrophobic graphite surfaces and found that the density of the IL was enhanced at the interfacial region. Kohler et al. studied the nature of the lamellar structure of 1-methyl-3-octylimidazolium tetrafluoroborate ([OMIM][BF_4_]) deposited on a solid aluminum substrate and found that liquid-like structures, coexisting liquid and solid phases, and solid-like structures might be formed.^3^ Jha et al. claimed that induced charges are the major contribution to adsorption of 1-ethyl-3-methylimidazolium ethyl sulfate ([EMIM][EtSO_4_]) on gold.^4^ The group of Smith^5,6^ simulated the behavior of 1-ethyl-3-methylimidazolium bis(trifluoromethylsulfonyl)imide ([EMIM][NTf_2_]) at the neutral sapphire interface and found that layering of cations and anions on the substrate was dominated by hydrogen bonding. The interactions of 1-butyl-3-methylimidazolium methyl sulfate ([BMIM][MeSO_4_]) with a hydroxylated silica surface was studied by Khaknejad et al., who reported preferential interactions of the imidazolium rings, which were coplanar with the silica surface.^7^

The interactions between ILs and surfaces determine their lubrication capacity, since the formation of a tribofilm, able to minimize the contact between the sliding surfaces, is crucial for good tribological performance. These films gradually develop until a steady state thickness, typically on the order of tens of nanometers.^8^ ILs have been tested as lubricants due to their specific properties, such as low vapor pressure, chemical and thermal stability, and electrical conductivity. In particular, ILs appear to be adequate lubricants of micro/nano-electromechanical systems (MEMS/NEMS) involving moving parts. Lubrication of these miniaturized devices has been the focus of intense research work in the last two decades^9−12^ because their large surface-to-volume ratios demand lubricants of high performance to avoid serious adhesion and friction problems. Various families of ILs based on the cations imidazolium, phosphonium, ammonium, pyrrolidinium, pyridinium, and guanidinium have been tested, both as neat lubricants or additives, in the lubrication of the silicon contacts, which mimic the behavior of MEMS and NEMS.^13−22^ The most successful anions were those containing sulfur-based functional groups.^21,22^

The capacity of protic ionic liquids (PILs) to lubricate silicon surfaces was recently investigated by our group.^23^ PILs are ionic liquids composed of Brønsted acids and bases, whose unique properties are conferred by the presence of proton-donor and proton-acceptor sites leading to dense hydrogen bonding. PILs are easily prepared by simple protonation of the cation using appropriate acids, and their price is lower compared to other ILs. The tribological behavior of several PILs based on the hydrogen sulfate ([HSO_4_]) and methyl sulfonate ([MeSO_3_]) anions, as additives to model lubricant poly(ethylene glycol) (PEG200), was compared to that of aprotic ILs, when applied to steel/silicon contacts, and the PILs outperformed the aprotic ILs in the reduction of friction. The PILs based on the cation 4-picolinium, [4-picH][HSO_4_] and [4-picH][MeSO_3_], presented a much better performance when compared to those based on the cation methylimidazolium, [MIMH][HSO_4_] and [MIMH][MeSO_3_]. The best additive was [4-picH][HSO_4_], which revealed an excellent lubrication capacity and minimized third-body abrasive wear.

**Scheme 1:**
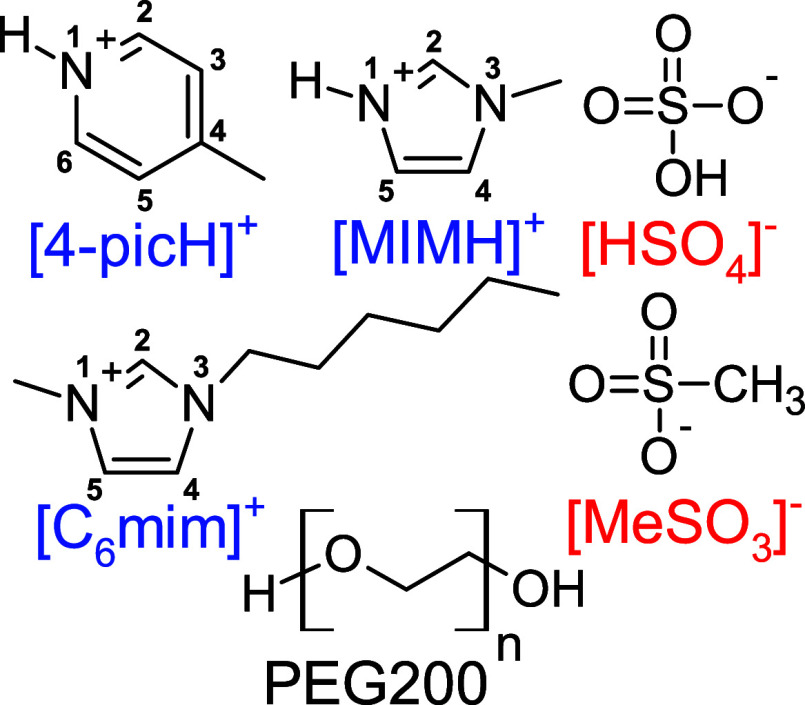
Molecular Structures of the Cations, Anions, and Solvent Used

In this work, we applied MD simulations to better understand the molecular behavior of six ILs added to PEG200 and their interaction with a silica surface. The investigated ILs include the PILs [4-picH][HSO_4_], [4-picH][MeSO_3_], [MIMH][HSO_4_], and [MIMH][MeSO_3_] and the non-PILs [C_6_mim][HSO_4_] and [C_6_mim][MeSO_3_]. The molecular structures of the cations and anions constituting the ILs are listed in Scheme 1. The choice of silica was done because silicon substrates kept at an ambient atmosphere oxidize spontaneously and are covered by a silicon oxide layer. The ultimate goal of this investigation was to explain the preferential interactions between the highest performance additives and the silica surface, which might justify the enhanced resistance of the adsorbed film to friction and wear. However, we must stress that the MD results do not encompass the formation of tribofilms at the interface nor possible differences in terms of chemical composition. Instead, our observations represent the first stages of the adsorption of the PILs and non-PIL additives at the glass surface.

## 2 Experimental Section

### 2.1 Materials

The reagents for the synthesis of the PILs were purchased and used without additional purification. The list of reagents is the following: 1-methyl-3-hexylimidazolium bromide >98% from Solchemar (Portugal), 4-methylpyridine 98% from Alfa Aesar, methanesulfonic acid 99% from Sigma-Aldrich, and sulfuric acid 95–97% and potassium hydrogen sulfate 99% from Merk. The solvents used were ethanol p.a. and distilled water. The resin used for the ionic exchange was Amberlyst A-26 (OH) from Merck. Poly(ethylene glycol) (MW 200), PEG200, was from Sigma-Aldrich, with water content <0.5%. Distilled and deionized water (DD) was obtained with a Millipore system.

Si substrates (squares, 1 × 1 cm^2^) were cut from Si b100N wafers (Si-Mat, Germany), with 0.5 mm of thickness, 1 nm of root-mean-square (RMS) roughness, and 1121–1428 HV of hardness. Si spheres (J. Hauser GMBH & Co., Germany) with 6 mm of diameter, 15 nm of RMS roughness, and 1412 HV of hardness were used as counter bodies.

### 2.2 Methods

The syntheses of the PILs are described in detail in our previous work.^23^ The syntheses of [C_6_mim][HSO_4_] and [C_6_mim][MeSO_3_] are described in the Supporting Information. In order to check the chemical structures and purities, the ionic liquids were characterized by ^1^H NMR (see Figure S1 in the SI). The tribological tests were done with a tribometer (TRB3, Anton Paar, Switzerland) in the configuration reciprocating ball-on-flat at room temperature (∼25 °C) and relative humidity (∼45%). The tribopairs were Si spheres/Si substrates. The sphere was placed on the tribometer arm, and the Si substrate was glued to a metallic container. Several drops of liquid were added to ensure full coverage of the surfaces. To assess the effect of the IL concentration, short tests (85 cycles, corresponding to 0.68 m of sliding distance) under low normal force of 1N and sliding speeds varying between 1 and 20 mm·s^–1^ were done. To study wear, longer tests (2375 cycles, corresponding to 19 m of sliding distance) were done under the normal load of 1N (Hertz contact stress of 584.5 MPa), at a constant speed of 8 mm·s^–1^. The amplitude of the reciprocal movement of the sphere was 4 mm. The results were analyzed using the software TriboX. The values of coefficient of friction (CoF) were obtained from the average of at least three results in independent experiments.

After the tribological tests, the Si substrates were carefully cleaned with acetone and dried with nitrogen to remove any traces of adsorbed material. The surfaces of the Si substrates were imaged using an optical profilometer (Profilm 3D, Filmetrics) and, for each track, the worn volume was estimated by multiplying the track length by the average of the cross-sectional areas of the worn track determined by numerical integration of the 2D profiles (3–5 measurements per track).

### 2.3 Molecular Dynamics Simulation Methodology

Molecular dynamics simulations were carried out with GROMACS 2020 and DL_POLY 2.20 packages^24−29^ from initial configurations generated by Packmol^30^ and fftool software.^31^ The ionic liquids and PEG200 (Table 1) were modeled with CL&P and OPLS-AA force fields. The parameters used in all MD simulations are given in Tables S1 and S6 in the Supporting Information (SI). The low-density initial cubic simulation boxes of the bulk phase of the ILs (with periodic boundary conditions in all directions) were subjected to a short MD stage consisting of 10^5^ steps of 2 fs duration in Nosé–Hoover *NpT* ensemble. Then, the MD boxes passed through successive simulated annealing stages (at least three) to release the internal constrains, following a later equilibration stage at 300 K. Finally, the production stage at 300 K and *p* = 1 atm used the Nosé–Hoover *NpT* ensemble (relaxation times of 0.5 ps for thermostat and 4.0 ps for barostat), velocity Verlet integration with a time step of 1 fs, distance cutoff of 1.60 nm, particle mesh Ewald with B-spline interpolation of order five, grid spacing of 0.10 for Fourier transforms (equilibration and production), and accuracy of Ewald sum kept at 5 × 10^–6^ at the cutoff (equilibration and production). The production stages were 10 ns long, with trajectories stored each for 2 ps. The final box size at *T* = 300 K and calculated densities of the bulk liquids are listed in Table S7 in the SI.

**Table 1:** Systems Studied at 300 K and the Type of Simulation Performed^a^

system	*n*_IL_	*n*_PEG200_	type
[4-picH][HSO_4_]	600:1200	0	bulk/interface
2% [4-picH][HSO_4_]^b^	20	1000	bulk/interface
5% [4-picH][HSO_4_]	50	1000	bulk
20% [4-picH][HSO_4_]	200	1000	bulk/interface
[4-picH][CH_3_SO_3_]	600:1200	0	bulk/interface
[MIMH][HSO_4_]	500:1200	0	bulk/interface
[MIMH][CH_3_SO_3_]	500:1200	0	bulk/interface
[C_6_mim][HSO_4_]	450:900	0	bulk/interface
[C_6_mim][CH_3_SO_3_]	900:900	0	bulk/interface

a The nIL and nPEG200 represent, respectively, the number of IL pairs and PEG200 units used in each system.

b Interface solid–liquid sampled with two independent MD runs.

The interactions in amorphous silica (SiO_2_) were described by a two-body Buckingham potential supplemented by a three-body truncated Vessal potential.^32^ The construction of the silica surface is described elsewhere^33^ and involved three steps: creation of the bulk box, cutting of the “dry” surface, and formation of the silanol groups. In brief, a cubic box with edges of 5.0 nm with a random distribution of the atoms at the desired composition was pre-equilibrated in *NVT* at 4000 K for 10 ps with a 1 fs time step, using a Nosé–Hoover thermostat with a coupling time constant of 0.5 ps. The density of the box was set to the corresponding experimental value of silicate glasses at room temperature.^32^ Then, the simulation box followed a temperature quench from 4000 to 300 K at a rate of 10 K·ps^–1^, which is the protocol for MD studies of glasses.^33,34^ Further annealing cycles between 300 and 1000 K were repeated. The “dry” silica surface is formed inserting a large gap in the *z* axis direction (the final *z* direction will have at least 5 times the *x*/*y* dimensions) and the surface was relaxed running simulated annealing cycles in *NVT*. To form the silanol groups at the glass surface, a layer of SPC^35^ water molecules was put over the glass surface, the box was heated at 1000 K under *NVT* for 100 ps, and then quenched to 300 at 10 K·ps^–1^. The distribution of the water molecules at the glass surface was evaluated, and the bonding parameters for the definition of the silanol groups at the interface followed the distance criteria *r*_Si-OW_ = 0.160 nm and *r*_O-HW_ = 0.100 nm (OW and HW refer to the O and H atoms of water, respectively). After, the excess (nonbonded) water was removed and the surface was equilibrated at 300 K by 200 ps. The final silanol surface density was in good agreement with the Kiselev–Zhuravlev constant.^36,37^

The IL–glass interface was constructed by inserting the equilibrated bulk box of the IL system over the treated glass slab. Eventual gaps between the IL and the glass were eliminated with 100 ps *NVT* runs at 350 K, with cutoff distances of 1.8 nm. The production runs were 20 ns long, with trajectories stored each for 4 ps. To speed up the MD simulations of the IL–glass interfaces, the glass slab was set frozen and the nonbonding interactions with the liquid phase were described by Coulomb and LJ potentials determined by the Lorentz–Berthelot mixing rules. The final box dimensions of the IL–glass systems were 5.0 × 5.0 × 65.0 nm^3^.

## 3 Results and Discussion

### 3.1 Tribological Tests

**Figure 1:**
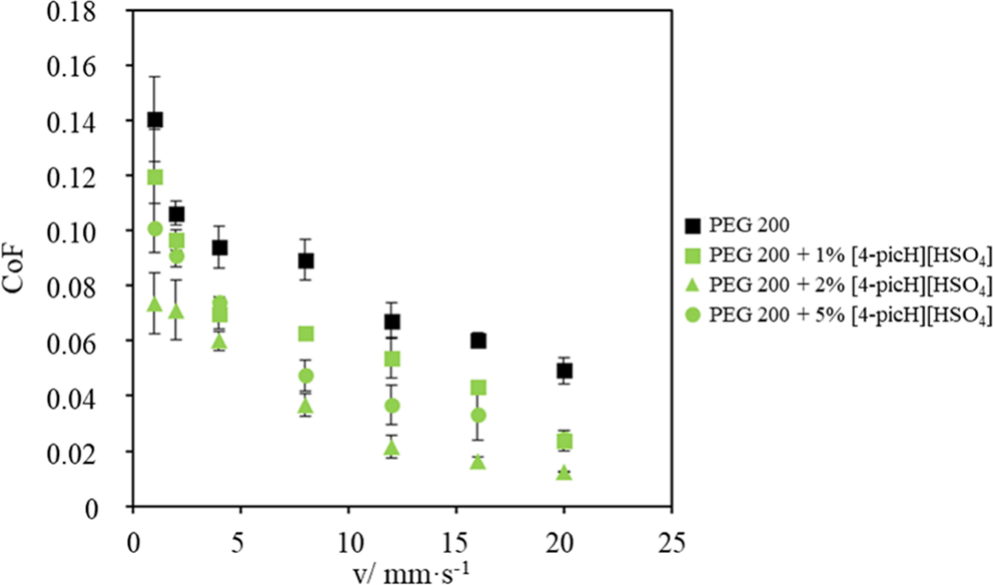
CoF vs sliding velocity obtained with neat PEG200 and the mixtures of [4-picH][HSO_4_] + PEG200 with different concentrations in tribological tests with Si/Si contacts under 1N. The errors are ± standard deviation (*n* ≥ 3).

The optimal concentration of the IL additives was determined by studying the effect of the amount of [4-picH][HSO_4_] added to PEG200 on the reduction of CoF of the tribological pair Si/Si under the load of 1N. Four different concentrations were tested: 0, 1, 2, and 5% (w/w). The results shown in Figure 1 demonstrate that CoF decreased in the presence of any concentration of [4-picH][HSO_4_], but 2% was the optimal value. The possible justification is that 1% was not sufficient to cover the whole surface with a lubrication film and the obtained CoF values were similar to those obtained with neat PEG200, mainly at low sliding velocities. On the other hand, the concentration of 5% led to the formation of a rougher film, which was not so efficient in the reduction of CoF.

**Figure 2:**
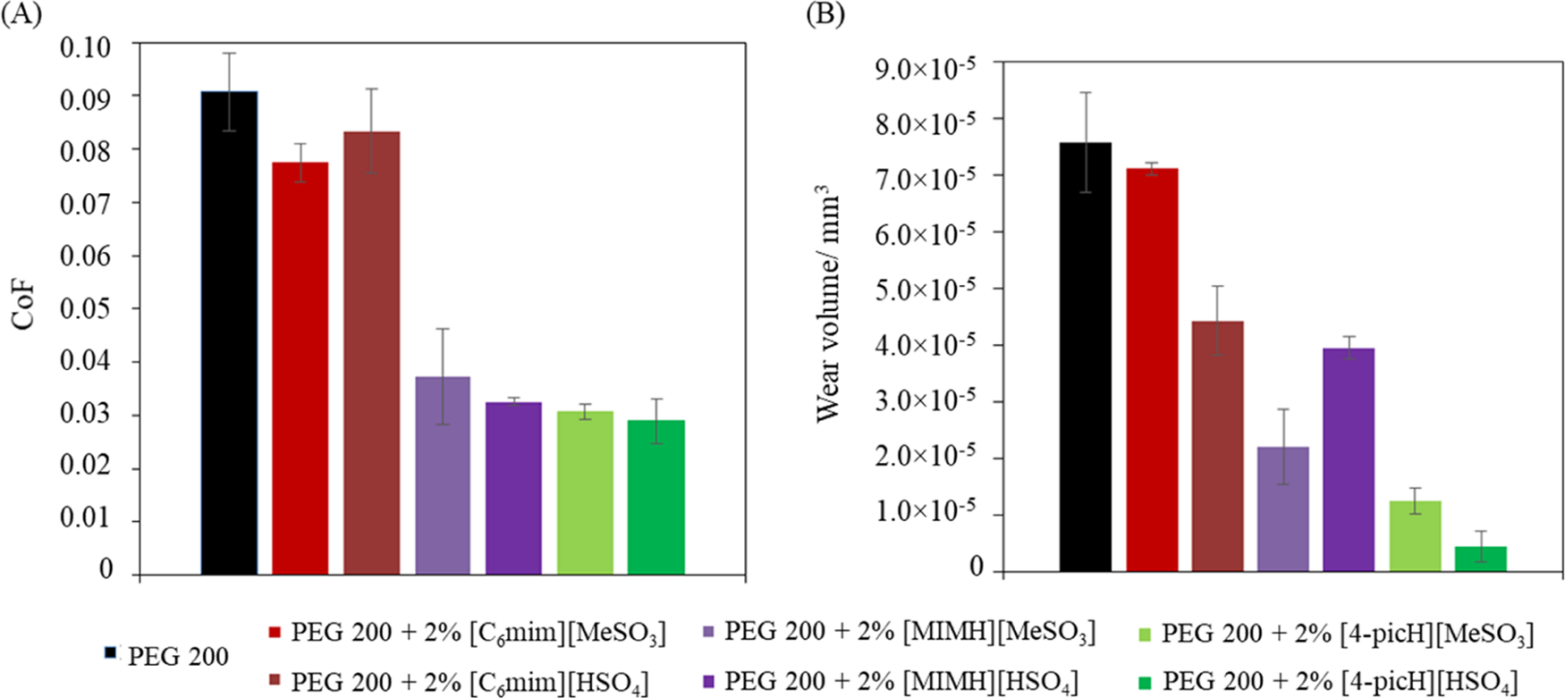
Results of the tribological tests using the mixtures PEG200 + 2% PIL as lubricants of Si/Si under 1N: (A) average CoF values and (B) average wear volumes. The errors are ± standard deviation (*n* ≥ 3). The values for the mixtures with [4-picH][HSO_4_] and [4-picH][MeSO_3_] were taken from a previous work.^23^

To compare the lubrication capacity of the six investigated ILs, long tribological tests (2375 s) were done under a load of 1N at a constant sliding velocity (8 mm·s^–1^). The average CoF values and the wear volumes of the Si substrates are presented in Figure 2A,B, respectively.

The tribological behavior seems to be essentially determined by the cation. The lowest CoF and wear were obtained in the presence of the additives based on the cation [4-picH]^+^, while the worst additives were those based on the cation [C_6_mim]^+^. According to our previous work,^23^ chemical and image analysis of the wear tracks on the Si substrates demonstrated that [4-picH][HSO_4_] is able to adsorb strongly on the silicon surface leading to the formation of a stable, protective film on the sliding surfaces. The preferential interaction of this PIL with the Si surface was suggested to be due to the synergy of the two ions: the symmetric cation [4-picH]^+^ interacted with the oxidized Si through hydrogen bonds with N–H and C–H of the picolinium ring; the [HSO_4_]^−^ anion interacted with the nonoxidized Si and the Si–O, through S–O and the hydroxyl groups, respectively.

### 3.2 Molecular Dynamics Simulations

#### 3.2.1 IL–Glass Interface

**Figure 3:**
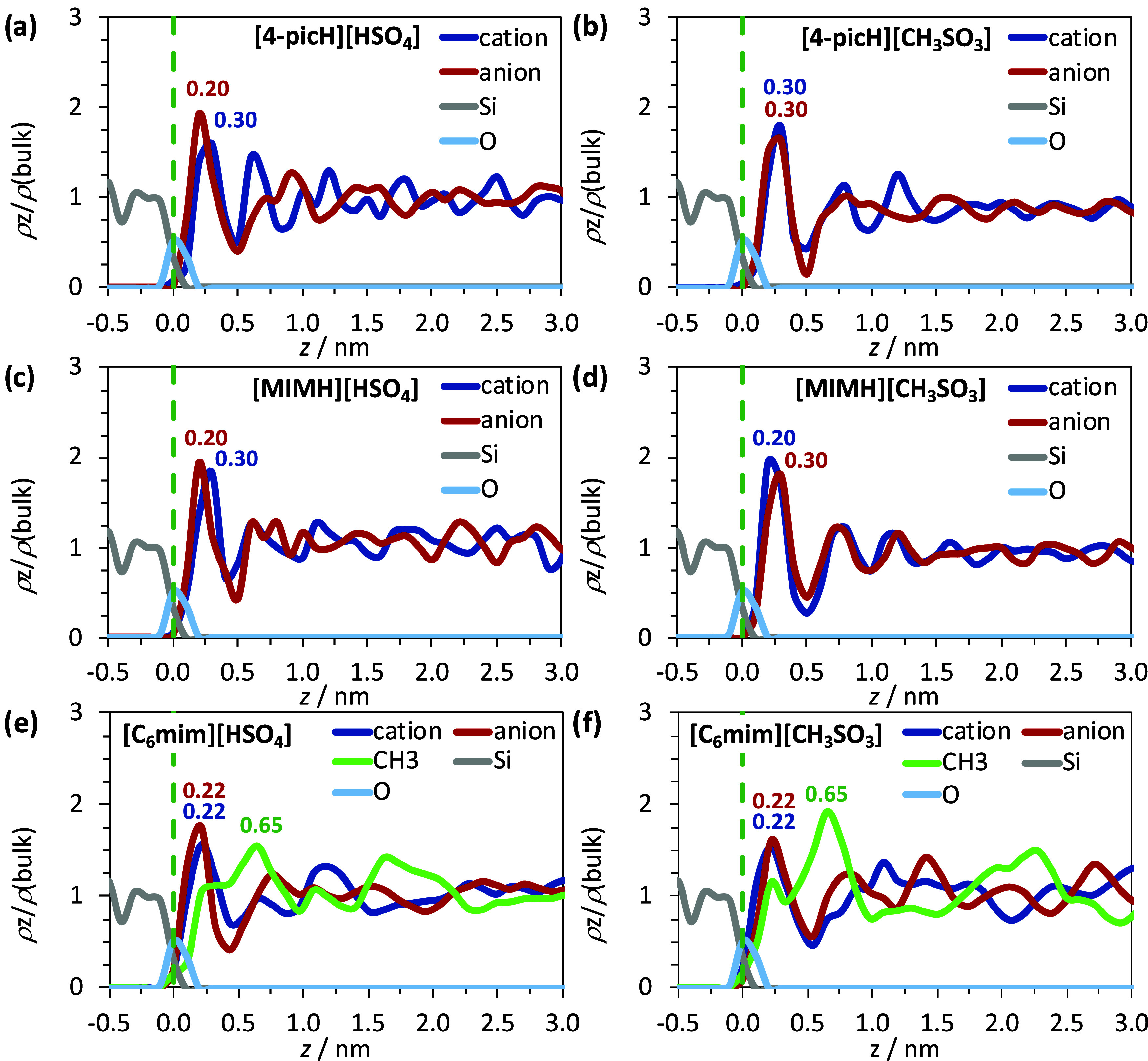
Number density profiles along the direction normal to the glass surface for the centers of mass of cations and anions of (a) [4-picH][HSO_4_], (b) [4-picH][CH_3_SO_3_], (c) [MIMH][HSO_4_], (d) [MIMH][CH_3_SO_3_], (e) [C_6_mim][HSO_4_], and (f) [C_6_mim][CH_3_SO_3_] (values normalized to nominal values in the case of a homogeneous isotropic bulk). The vertical dashed line represents *z* = 0 nm and is defined as the outermost atoms at the glass surface (O atoms of silanol groups, represented in light blue). The gray line denotes the Si atoms in the solid substrate.

MD simulations at *T* = 300 K of the pure ILs in contact with the silica glass surface were done before studying the mixtures with PEG200. The snapshots of the MD simulations of the ILs at the amorphous silica interface are presented in Scheme S1. The [4-picH]^+^ and [MIMH]^+^ ions do not have nonpolar side chains attached to the picolinium or imidazolium ring moieties, and their pairing with anions such as [HSO_4_]^−^ or [CH_3_SO_3_]^−^ results in ILs with only polar domains. On the other hand, the ILs with the [C_6_mim] cation exhibit the nonpolar domains associated with the presence of the alkyl chain. Figure 3 shows the density profiles along the direction normal to the glass surface of the centers of mass of ions in [4-picH][HSO_4_], [4-picH][CH_3_SO_3_], [MIMH][HSO_4_], [MIMH][CH_3_SO_3_], [C_6_mim][HSO_4_], and [C_6_mim][CH_3_SO_3_] at 300 K. A general feature observed is the enhanced IL density in the interfacial region. The first IL layer (ca. 0.5 nm) in close contact with the glass interface is more affected by the presence of the solid substrate, with a quick decay of the density oscillations to the fluid-like distribution after 1.5 nm in all cases (about three IL layers). Since both cations and both anions can perform H bonds with silanol groups at the glass surface, the first peak maxima are nearly the same distance for all species. The periodicity of the layers is consistent with experimental evaluations for imidazolium-based ILs, in the range 0.4–0.7 nm,^38,39^ indicating that the picolinium- and imidazolium-based ILs under study have equivalent physical dimensions and are subjected to similar packing. Also, the numeric density profiles reveal that the [HSO_4_]^−^ is closer to the glass surface than the bulkier [4-picH]^+^, [MIMH]^+^, and [C_6_mim]^+^ ions, in line with previous MD studies involving large cations and small anions ([C_4_mim][PF_6_] and [C_4_mim][BF_4_]) near amorphous silica interfaces.^40^ The [CH_3_SO_3_]^−^ ion is slightly closer to the amorphous interface than the [4-picH]^+^ cation as well, but the inverse situation is observed for both imidazolium-based cations [C_6_mim]^+^ and [MIMH]^+^. The density profiles of the terminal group of the alkyl chain of the [C_6_mim]-based ILs are seen in Figure 3e,f. These profiles gauge the location of the nonpolar domains as a function of the distance to the interface, and in both cases, the corresponding profile of the CH_3_ group is in phase opposition with the density profiles of the polar part. Also, for both [C_6_mim]-based ILs, the maximum of the nonpolar domain appears at 0.65 nm, with a shoulder at 0.22 nm. Together, these two pieces of information indicate that in both ionic liquids, the imidazolium ring is facing the glass surface, while the aliphatic chain is preferentially oriented toward the bulk. The surface area per ion pair was calculated by integrating the numerical density profiles between *z* = 0 nm (glass surface) and *z* = 0.5 nm (first minima in the density profiles) and is presented in Table S8 (SI). In the case of pure ILs in contact with the amorphous substrate, the results indicate that the [CH_3_SO_3_]^−^-based ILs occupy larger areas at the silicate interface when compared with the [HSO_4_]^−^ anion and that the alkyl chain in [C_6_mim]^+^ has a pronounced effect in reducing the number of ions present at the interface.

**Figure 4:**
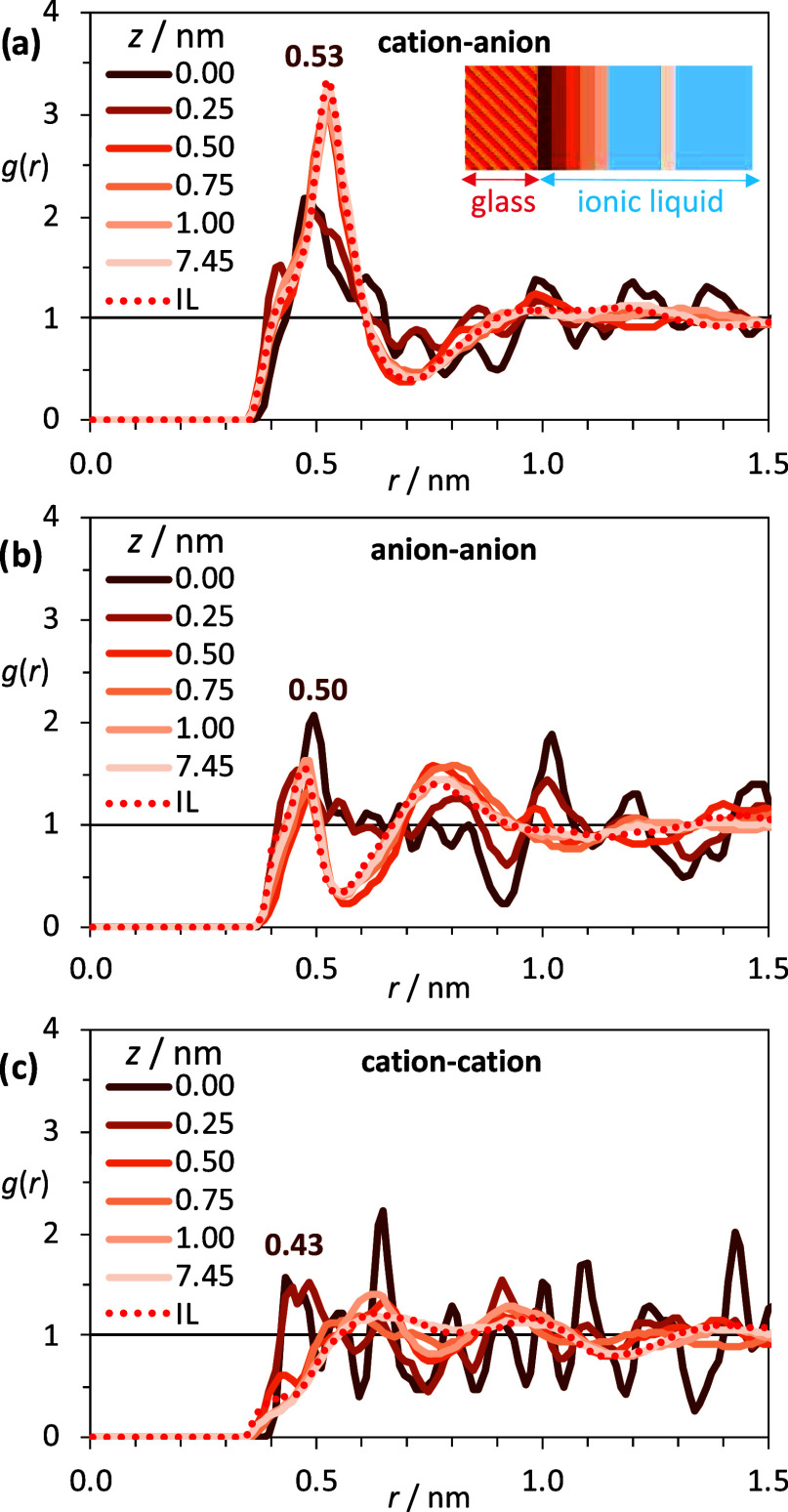
Tangential RDFs between the centers of mass of (a) cations and anions, (b) anions, and (c) cations for the [4-picH][HSO_4_] protic ionic liquid, collected at several *z* distances (in nm) from the glass interface. The dotted line represents the corresponding RDF of the isotropic ionic liquid. The inset shows approximately where the TRDF layers (continuous vertical lines) were collected with respect to the dimensions of the simulation box, while the glass substrate is depicted as diagonal lines.

The glass surface has regions with different densities of silanol groups and is uneven in terms of the local charge distribution (resulting in an inhomogeneous electrical field). Thus, the glass interface does not only induce structural layering of the ILs but also leads to charge and density fluctuations within those planes.^33^ To understand the orientation of cations and anions present in the first layer in contact with the silica interface, we carried out tangential radial distribution functions (TRDFs) between selected centers on the glass surface and some functional groups of cations and anions. The TRDFs were determined in 0.5 nm thick layers at several *z* distances of the glass interface, with *z* = 0 defined as the outermost atoms of the glass surface. The TRDFs between the centers of mass of cations and anions of [4-picH][HSO_4_] are presented in Figure 4 together with the RDFs of the neat ILs (the corresponding TRDFs for PIL [4-picH][CH_3_SO_3_] are shown in Figure S2 in the SI). The local structuration of cations and anions in the first IL layer is quite distinctive, while the distribution of the other layers resembles more that seen in the isotropic IL phase. In the vicinity of the uncharged silicate substrate interface, the TRDFs cation–anion and cation–cation reveal that cations and anions are closely packed and that the tridimensional polar network of the ILs is partially lost.^33^ The morphological differences among the anions are noticeable in the first peak of the TRDFs anion–anion. The [CH_3_SO_3_]^−^ exhibits some preferential orientation toward the glass surface that reduces the electrostatic repulsions and allows close approximation between anions present in the first layer, resulting in interionic distances ca. 0.15 nm smaller than the neat IL. The opposite trend is observed for the quasi-spherical [HSO_4_]^−^, where the preferred orientation facing the solid substrate slightly increases the interionic distances to 0.50 nm at the glass interface, in comparison with 0.44 nm observed in the isotropic PIL. For the glass–IL systems [MIMH][HSO_4_], [MIMH][CH_3_SO_3_], [C_6_mim][HSO_4_], and [C_6_mim][CH_3_SO_3_], the TRDFs are depicted respectively in Figures S3–S6 in the SI. The same overall trends were observed in those systems and the discussion does not need to be further extended.

To further evaluate the organization of the first layer of the ILs in contact with the amorphous silica substrate, the tangential radial distribution functions (TRDFs) between the oxygen atom of the silica glass (SiO_2_) and the H atoms of the cation ring were determined for a 0.5 nm thick layer of IL at the glass interface (centered at *z* = 0). In the case of the cations, the most acidic hydrogen in [4-picH]^+^ and [MIMH]^+^ is bonded to the N atom of the ring, while in the [C_6_mim]^+^ ion, it is bonded to C2 carbon, located between the two nitrogen atoms of the imidazolium ring. First, Figure S7 in the SI compares the TRDFs of the oxygen atom of the SiO_2_ group and all H atoms in the imidazolium-based cations [C_6_mim]^+^ and [MIMH]^+^, with [HSO_4_]^−^ (Figure S7a) and [CH_3_SO_3_]^−^ (Figure S7b) as counterions. The alkyl groups in N1 and N3 do not allow a close approach of [C_6_mim]^+^ to the interface, and H atoms at C2, C4, and C5 are equally distant to the glass surface (ca. 0.22 nm). For [MIMH]^+^, the preferential orientation of the H atom at N1 (N1–H) toward the surface is clear. The anion size is also an important aspect in the arrangement of the [C_6_mim]^+^ ion near the interface. The accommodation of the larger anion [CH_3_SO_3_]^−^ in the first layer breaks the weakest specific interactions of the cation with SiO_2_ groups (namely, H atoms at C4 and C5) and slightly promotes H bonding at C2, in comparison to [HSO_4_]^−^. Thus, the comparison between [C_6_mim]^+^ and [MIMH]^+^ indicates that the interactions of the cation with the surface as well as its orientation are greatly influenced by the presence of alkyl substituents or large anions. The TRDFs determined for a 0.5 nm thick layer of IL at the glass interface between the oxygen atom of the SiO_2_ group and the most acidic hydrogen atom of the cation (N1) in ILs [4-picH][HSO_4_] and [4-picH][CH_3_SO_3_] are shown in Figure S8 in the SI. In order to provide a comparative analysis, the results for N1–H of [MIMH][HSO_4_] and [MIMH][CH_3_SO_3_] are displayed in Figure S8 as well. The organization is remarkably similar to a sharp peak at ca. 0.14 nm and with both cations anchored to the surface by the acidic H atoms, while [HSO_4_]^−^ and [CH_3_SO_3_]^−^ ions show little influence.

**Figure 5:**
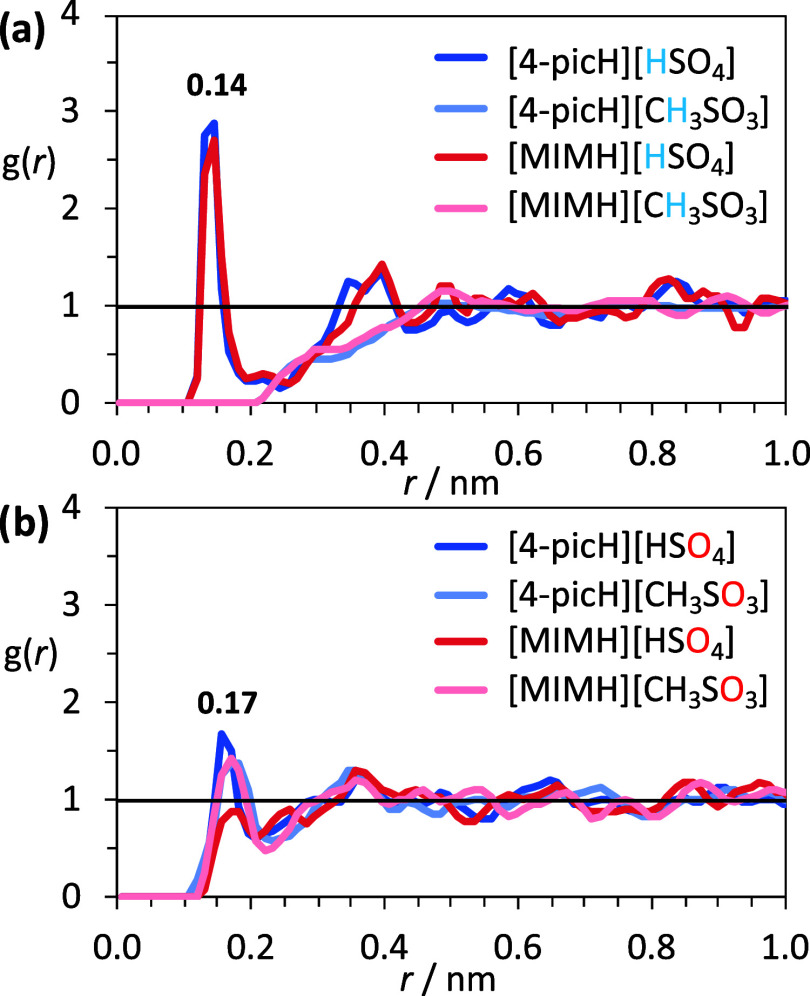
Tangential RDFs at *z* = 0 nm between (a) the H atoms of the anions ([4-picH][HSO_4_], [4-picH][CH_3_SO_3_], [MIMH][HSO_4_], and [MIMH][CH_3_SO_3_]) and the O atoms of the SiO_2_ groups in the glass surface and (b) between O atoms of the anion ([4-picH][HSO_4_], [4-picH][CH_3_SO_3_], [MIMH][HSO_4_], and [MIMH][CH_3_SO_3_]) and the H atom of the silanol groups (SiOH).

The TRDFs between the H atom of the anion and the O atom of the SiO_2_ groups at the interface are presented in Figure 5a for [4-picH][HSO_4_], [4-picH][CH_3_SO_3_], [MIMH][HSO_4_], and [MIMH][CH_3_SO_3_]. Interestingly, the functions indicated that the H atom of the [HSO_4_]^−^ anions located in the first contact layer are facing the glass surface, with a peak maximum at ca. 0.14 nm and a second less pronounced peak at ca. 0.40 nm. On the other hand, there is no indication of preferential orientation of the methyl H atoms of the [CH_3_SO_3_]^−^ toward the glass interface, with the TRDFs quickly evolving to the isotropic distribution. The morphology of the cation does not affect the organization of the H atoms of the anions in the vicinity of the glass interface. Figure 5b depicts the TRDFs between the O atoms of the anion ([4-picH][HSO_4_], [4-picH][CH_3_SO_3_], [MIMH][HSO_4_], and [MIMH][CH_3_SO_3_]) and the H atom of the silanol groups (SiOH) present at the surface. The profiles of the TRDFs show a peak maximum near 0.17 nm and then a fast development to the isotropic distribution. In terms of selectivity, however, the TRDF peaks show subtle differences among the PILs. Paired with the picolinium-based cation, the [HSO_4_]^−^ ion can orient its O atoms to the silanol groups. In the case of the [MIMH]^+^ cation, the O atoms of the anion are less susceptible to performing specific interactions with the silanol at the glass surface because there is a competition between SiOH groups and the multiple acidic H atoms available in the imidazolium ring moiety able to engage in H bonds with the anion.

Also, it is possible to perform a rough estimation of the tilt angle of the cation ring axis with respect to the surface normal using inverse trigonometric functions and the interatomic distances evaluated within the framework of the employed force field and with the number density profiles of some key atom types (not shown). In the case of the [MIMH]^+^ ion, an axis running parallel to the aromatic ring was defined between the H atom at N1 and the methyl group at N3, giving an OPLS length of ca. 0.467 nm for this axis. For [4-picH]^+^, an OPLS distance of ca. 0.529 nm was defined for the axis passing through the H atom at N1 and the 4-methyl group. The tilt angle for [4-picH]^+^ and [MIMH]^+^ in the first layer is estimated in 30°. For the [C_6_mim]^+^ ion, the OPLS distance of the axis passing by the H atoms at C2 and C4 (or C2 and C5) is ca. 0.445 nm. In this last case, because of the presence of the methyl and hexyl groups at N1 and N3, the imidazolium ring is tilted about 65° with respect to the surface normal.

The orientational ordering parameter *S* gives the orientation of two vectors and is defined as the average second Legendre polynomial^41^
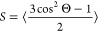
1where Θ is the angle between a reference axis (defined here as the normal to the glass surface) and the direction vectors outlined in Figure S9 in the SI. The ring orientational ordering parameters are obtained as a time average of Θ. Thus, the angles 0° (*S* = 1) and 90° (*S* = −0.5) symbolize perfectly aligned and perfectly perpendicular axes with respect to the surface normal, respectively. The angle of 54.7° (*S* = 0) corresponds to an isotropic distribution or a system perfectly oriented at the magic angle. The ring orientational ordering parameters of the ILs as a function of the distance to the surface are displayed in Figure S10 in the SI and are in line with the estimations from the tilt angles. In these figures, the ordering parameters of the cations paired with [HSO_4_]^−^ and [CH_3_SO_3_]^−^ anions are portrayed for the two direction vectors defined (NC/NN and CC). Between 0.5 and 1 nm (1 and 2 IL layers), the ring orientational parameters evolve to isotropic distributions in all cases. Surprisingly, the [MIMH]^+^ ions very close to the glass surface (*z* < 0.2 nm) are tilted as the [C_6_mim]^+^, but for larger *z*, the profiles resemble that of [4-picH]^+^, with CC axis exhibiting more tendency to be perpendicular to the surface normal than NC or NN axis. In comparison with [HSO_4_]^−^, the results suggest that [CH_3_SO_3_]^−^ slightly enhances the vector orientation already dominant in contact with the surface for the imidazolium-based cations, while [4-picH]^+^ seems to be little affected on its orientation by the change of the anion.

At this point, it is relevant to discuss the interesting interplay of van der Waals (1–10 *k*_B_*T*) and H bonding (10–20 *k*_B_*T*) intermolecular noncovalent interactions holding the cations at the glass interface. In the vicinity of silica 001 surface (the most stable crystallographic plane of α-quartz), the imidazolium ring in [C_4_mim][CH_3_SO_3_] is coplanar to the surface, suggesting that the cation π^+^ interactions with the surface atoms are maximized.^7^ On the other hand, this orientation is unfavorable to H bonding (in terms of angle criterium for H bond interactions) through H atoms at C2, C4, and C5. The [C_6_mim]^+^ cation, however, is oriented in such a way that the H bonds are weak (by distance criterion), as well as the cation π^+^ interactions. The [4-picH]^+^ and [MIMH]^+^ fulfill the distance and angle criteria for strong to moderate H bonding of the N–H groups (as well as for moderate to weak H bonds with neighboring C–H) with the surface but have little engagement of cation π^+^ interactions because of the unfavorable angle between the ring normal and the glass surface. In the vicinity of the glass surface, the imidazolium-based cations tends to flip over and orient to the surface the multiple sites able to perform H bonds.

#### 3.2.2 Glass Interface with Mixtures PEG200 + [4-picH][HSO_4_]

**Figure 6:**
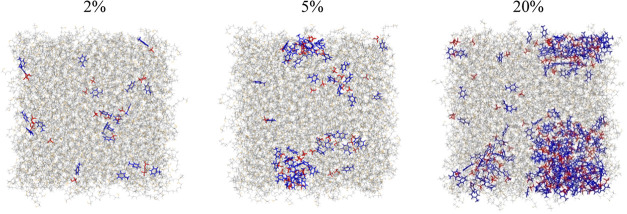
Snapshots of the MD simulations of the mixtures of PEG200 with the PIL [4-picH][HSO_4_] at concentrations 2%, 5%, and 20% at *T* = 300 K. The blue and red colors represent cations and anions, respectively, while PEG200 is depicted in gray.

The MD results for the ILs near the amorphous silica interface have indicated that there are preferential interactions between [4-picH][HSO_4_] and the glass surface. Also, the experimental data revealed good tribological performance of [4-picH][HSO_4_] as an additive in PEG200 to lubricate silicon/silicon contacts as well as steel/silicon pairs.^23^ First, the snapshots of the MD simulations of the bulk mixtures of PEG200 with [4-picH][HSO_4_] at concentrations 2, 5, and 20% (w/w) are presented in Figure 6 and the aggregate probability distribution functions for the corresponding systems are depicted in Figure S11 in the SI. At 2%, the PIL is dispersed at random inside the PEG200 phase and ca. 12% of the cations and anions are found isolated. The aggregate population with the highest probability is composed of 2 ion pairs. The mixture with 5% of [4-picH][HSO_4_] exhibits the formation of aggregates with 31 ion pairs, representing ca. 60% of all ions present in the mixture, suggesting that the IL is already beyond its PEG-solubility. In the PEG200 + 20% [4-picH][HSO_4_] system, there is the formation of a large aggregate with more than 80% of the ions in solution, clearly indicating phase separation. Interestingly, when used as lubricant additives to PEG200, amino acid-based ILs exhibited highest CoF reduction also at 2% (w/w) IL concentration,^42^ while imidazolium-based ILs presented the best tribological behavior both at 2 and 5 (w/w) IL concentration.^22^ Considering the experimental CoF values presented in Figure 1 and the MD results discussed so far, the simulations near glass interfaces for PEG200 + [4-picH][HSO_4_] systems were carried out only for the mixture containing 2% [4-picH][HSO_4_] as the additive.

The snapshots of the MD simulations of the pure PEG200 and PEG200 + 2% [4-picH][HSO_4_] in contact with the silicate interface are presented respectively in Schemes S2 and S3 in the SI. As stated before, two independent MD runs were carried out for the PEG200 + 2% [4-picH][HSO_4_] system, with both configurations evolving to the same PIL surface coverage after equilibration. The snapshots and data in Table S8 in the SI indicate that only a fraction of the PIL effectively covers the amorphous interface in the first layer (ca. 4% of the glass surface is covered by ion pairs), while the largest portion of the additive is dispersed in the bulk. We also run an MD simulation for the more concentrated system PEG200 + 20% [4-picH][HSO_4_], and even in this case, the glass surface is only 15% covered by IL pairs. These results suggest that the molecular origin of the enhanced lubricating performance of PEG200-PIL systems is not solely an effect of the PIL adsorbed at the interface, but it arises from structural modifications in the adsorbed layer induced by the combination of PILs with PEG200. The classical MD simulations performed here represent the first stages of the adsorption of the PILs and non-PIL additives at the glass surface. The subsequent formation of protective tribofilms at the lubricating interface upon rubbing and differences regarding chemical composition or surface protection were not accessed by our calculations.

**Figure 7:**
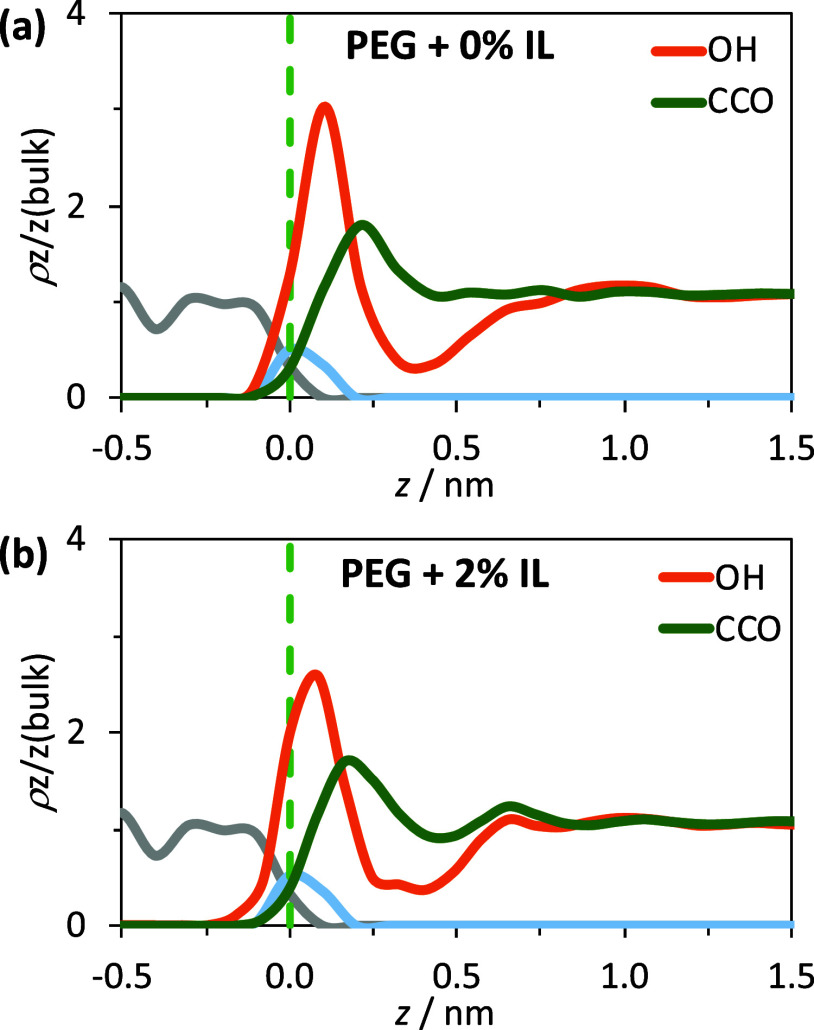
Number density profiles along the direction normal to glass surface for OH groups and CH_2_CH_2_O units of (a) PEG200 and (b) PEG200 + 2% [4-picH][HSO_4_] (values normalized to nominal values in the case of a homogeneous isotropic bulk). The vertical dashed line represents *z* = 0 nm and is defined as the outermost atoms at the glass surface (O atoms of silanol groups, represented in light blue). The gray line denotes the Si atoms in the solid substrate.

The density profiles along the normal to the surface for PEG200 and PEG200 + 2% [4-picH][HSO_4_] are depicted in Figure 7. For better visualization, the profiles of PEG200 are discriminated in OH groups and ethylene oxide segments, indicating that the OH groups are facing the glass interface (Figure S12 in the SI portrays the same data discriminated by atom types). The substrate surface induces a very weak structural layering of PEG200, and the thickness of the first layer is ca. 0.4 nm. Beyond the first well-defined strongly adsorbed layer, the structure of the pure molecular solvent becomes featureless because of the nonexistence of electrostatic interactions, in clear opposition to what is usually observed for ILs. However, the addition of 2% [4-picH][HSO_4_] is enough to induce a significant change in the organization of the model lubricant near the glass interface, clearly imposing some ordering until ca. 0.8 nm outside the first layer. The inspection of Figures 7 and S12 indicates that the distance between PEG200 molecules and the glass surface decreases when 2% (w/w) [4-picH][HSO_4_] is present. In other words, the presence of the 2% of PIL leads to the formation of a compact protective film on the surface,^23^ corroborated by the reduction in CoF and wear in Figure 2. In close contact with the glass substrate, the OH groups of pure PEG200 can perform H bonds with neighboring PEG200 molecules, as seen on TRDFs presented in Figure S13a,b in the SI. The presence of small amounts of [4-picH][HSO_4_] breaks the already faint hydrogen bonding network among PEG200 chains adsorbed at the glass surface (Figure S13c,d in the SI) but does not disrupt the H bonding of PEG200 with SiO_2_ groups (Figure S14 in the SI).

In terms of the intramolecular distance between OH groups in PEG200, the 2% addition of PIL leads to a marginal increase in the OH–OH distances of the molecules in contact with the glass surface (Figure S15 in the SI). As the distance to surface increases and the PEG200 molecules have more freedom to adopt bent conformations, the intramolecular OH–OH distance decreases and it is insensitive to the presence of the additive. The torsion angle distributions of the PEG200 chain are depicted in Figure S16 in the SI. There, ϕ_1_ represents the dihedral angle –O(R)–C–C–O(R)– and ϕ_2_ denotes the torsion angle –O(R)–C–C–O(H), with the *trans* conformation corresponding to ϕ*_n_* = 180°. For both ϕ_1_ and ϕ_2_, the population of *gauche* conformers increases with the distance to the interface (Figure S16a,b), in line with the reduction seen in intramolecular OH–OH distances. The torsion angles exhibit the same behavior with the addition of the PIL (Figure S16a,b). However, the formation of a more compact protective film adsorbed at the surface in the presence of [4-picH][HSO_4_] is revealed by a larger *trans* population of ϕ_1_, in comparison to the same distribution for pure PEG200.

## 4 Conclusions

The electrostatic forces present at the glass interface play an important role in the adsorption mechanism since glass and crystalline SiO_2_ inherently carry negative charges on their surface. For example, when silicate glass surfaces are immersed in water, they develop negative surface charge density due to the dissociation of the silanol groups at the interface. Additionally, the unevenly distributed electric field at glass surfaces can induce density fluctuations in pure ILs up to 3 nm away from the interface.^33^ In other words, the ionic liquids respond to the surface charge pattern imposed by the glass interface. In this work, we compared the interfacial properties of the ionic liquids [4-picH][HSO_4_], [4-picH][CH_3_SO_3_], [MIMH][HSO_4_], [MIMH][CH_3_SO_3_], [C_6_mim][HSO_4_], and [C_6_mim][CH_3_SO_3_] at the silica surface, in order to better understand their behavior as lubricant additives in PEG200. All of the six ILs were subjected to the same negative surface potential since the glass slab was the same in all cases. Once this initial step in adsorption, driven by electrostatic forces, took place, other stages begin to develop. A common feature to all studied IL–glass systems pointed out by the MD simulations is that the first adsorbed IL layer is ca. 0.5 nm thick. The balance between van der Waals and H bonding interactions holding the cations at the glass interface play a central role in the tribological properties of the ionic liquids. Small differences in the cation functionalization can lead to a significant change in their lubricant properties. The average CoF and wear volumes of silicon substrates are smaller for [4-picH]-based PIL additives than for [MIMH] and [C_6_mim]-based ionic liquids. Regarding the anions, the MD results on pure ILs on the solid substrates indicated that the weak specific interactions of the cation with the glass surface are lost to accommodate the larger anion in the first contact layer. This is more evident in cations such as [C_6_mim]^+^ where the presence of alkyl groups already conditioned the orientation of the cation near the surface.

Additionally, our findings suggest that the enhanced lubrication performance of PEG200 with [4-picH][HSO_4_] cannot be exclusively attributed to the presence of the PIL at the interface since only a fraction of the PIL is effectively adsorbed at the silica surface. Instead, it arises from synergistic interactions between the PIL and PEG200 at the adsorbed layer. The small amounts of [4-picH][HSO_4_] disrupts the already faint hydrogen bonding network between PEG200 chains adsorbed at the glass surface, while leaving the H bonding between PEG200 and SiO_2_ groups intact.

## Supporting Information Available

Syntheses and ^1^H NMR spectra of the PILs [C_6_mim][MeSO_3_] and [C_6_mim][HSO_4_]; snapshots of the MD simulations at 300 K for interfaces IL–glass, PEG200–glass, and IL/PEG200–glass; tables with force field parameters of the MD simulations, final box length, calculated densities at 300 K, and surface areas per ion pair (or per PEG200 molecule) at the glass interface; tangential RDFs between selected centers in pure ILs, pure PEG200 and PEG200 + 2% [4-picH][HSO_4_] as a function of the vertical distance from the glass interface; ring orientational ordering parameter as a function of the distance to the glass surface; probability distribution function of cation–anion aggregates in PEG200 + [4-picH][HSO_4_] systems as a function of the total % of ions; number density profiles along the direction normal to glass surface for OH groups in PEG200 and PEG200 + 2% [4-picH][HSO_4_]; intramolecular distance distribution functions of the OH groups in PEG200 and PEG200 + 2% [4-picH][HSO_4_]; torsion angle distributions of PEG200 chains as a function of distance from the glass interface (PDF)
