# Supplementary material for: Molecular Interactions between Ionic Liquid Lubricants and Silica Surfaces: An MD Simulation Study
Source: J Phys Chem B. 2024 Mar 5;128(10):2559–68. doi: 10.1021/acs.jpcb.3c08397 (PMC10945478; doi:10.1021/acs.jpcb.3c08397)
Supplement: Supplementary file 1 — jp3c08397_si_001.pdf [file jp3c08397_si_001.pdf]

Supporting Information for

Molecular Interactions Between Ionic Liquid Lubricants and Silica Surface: a MD Simulation Study

Mariana T. Donato[1,2], Rogério Colaço[3], Luis C. Branco[2], Benilde Saramago[1], José N. Canongia Lopes[1], Karina Shimizu[1], Adilson Alves de Freitas[1]**

[1] Centro de Química Estrutural, Institute of Molecular Sciences, Departamento de Engenharia Química, Instituto Superior Técnico, Universidade de Lisboa, Av. Rovisco Pais 1049 001 Lisboa, Portugal

[2] LAQV-REQUIMTE, Departamento de Química, NOVA School of Science and Technology, Universidade NOVA de Lisboa, Campus da Caparica, 2829-516 Caparica, Portugal

[3] IDMEC-Instituto de Engenharia Mecânica, Departamento de Engenharia Mecânica, Instituto Superior Técnico, Universidade de Lisboa, Av. Rovisco Pais, 1049-001 Lisboa, Portugal

Syntheses of the PILs:

1-Methyl-3-hexylimidazolium mesylate: [C_6mim][$MeSO_3$]

1.2 mL of 1-methyl-3-hexylimidazolium bromide and 1 equivalent of anionic resin Amberlyst A-26 (OH) (~1.5 g) were added to a 50 mL round-bottom flask, with 20 mL of ethanol. The mixture was stirred for 30 minutes at room temperature and, after that, 0.4 mL of methanesulfonic acid in ethanol were added. The mixture was stirred for 24h at room temperature. The solvent was evaporated and the final product was dried in vacuum and obtained as a viscous yellow liquid (1.61 g, η = 78%).

^{1}H-NMR (δ, $CDCl_3$, 400 MHz): 9.63 (s, 1H), 7.39 (s, 1H), 7.30 (s, 1H), 4.25 (t, 2H, J = 8.0 Hz), 4.04 (s, 3H), 2.91 (s, 3H), 1.92 – 1.84 (m, 2H), 1.37 – 1.22 (m, 6H), 0.87 (t, 3H, J = 4.0 Hz) ppm.

1-Methyl-3-Hexylimidazolium hydrogen sulfate: [C_6mim][HSO_4]

0.65 mL of 1-methyl-3-hexylimidazolium bromide and 0.48g of potassium hydrogen sulfate were dissolved in water and added to a 50 mL round-bottom flask. The mixture was stirred for 24h at room temperature. The solvent was evaporated and the final product was dried in vacuum and obtained as a viscous yellow liquid (0.84 g, η = 66%).

^{1}H-NMR (δ, $CDCl_3$, 400 MHz): 10.30 (s, 1H), 7.39 (t, 1H, J = 4.0 Hz), 7.29 (t, 1H), 4.75 (s, 1H), 4.30 (t, 2H, J = 8.0 Hz), 4.10 (s, 3H), 1.94 – 1.87 (m, 2H), 1.38 – 1.26 (m, 6H), 0.87 (t, 3H, J = 8.0 Hz) ppm.

^{1}H-NMR [C_6mim][$MeSO_3$]

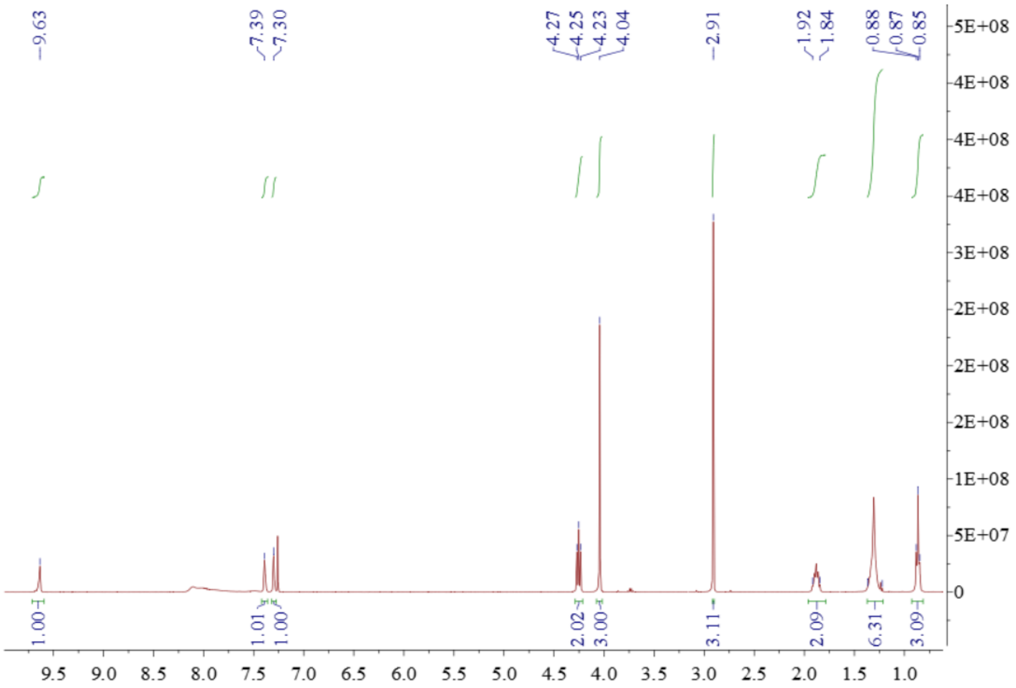

^{1}H-NMR [C_6mim][HSO_4]

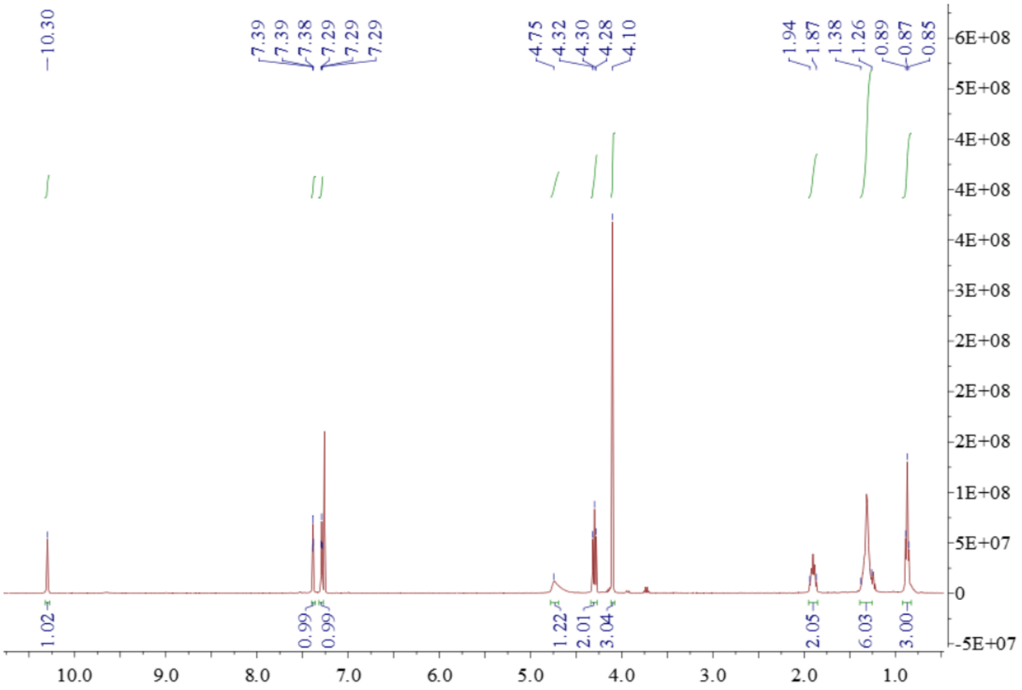

Figure S1. ^{1}H-NMR of [C_6mim][$MeSO_3$] (top) and [C_6mim][HSO_4] (bottom).

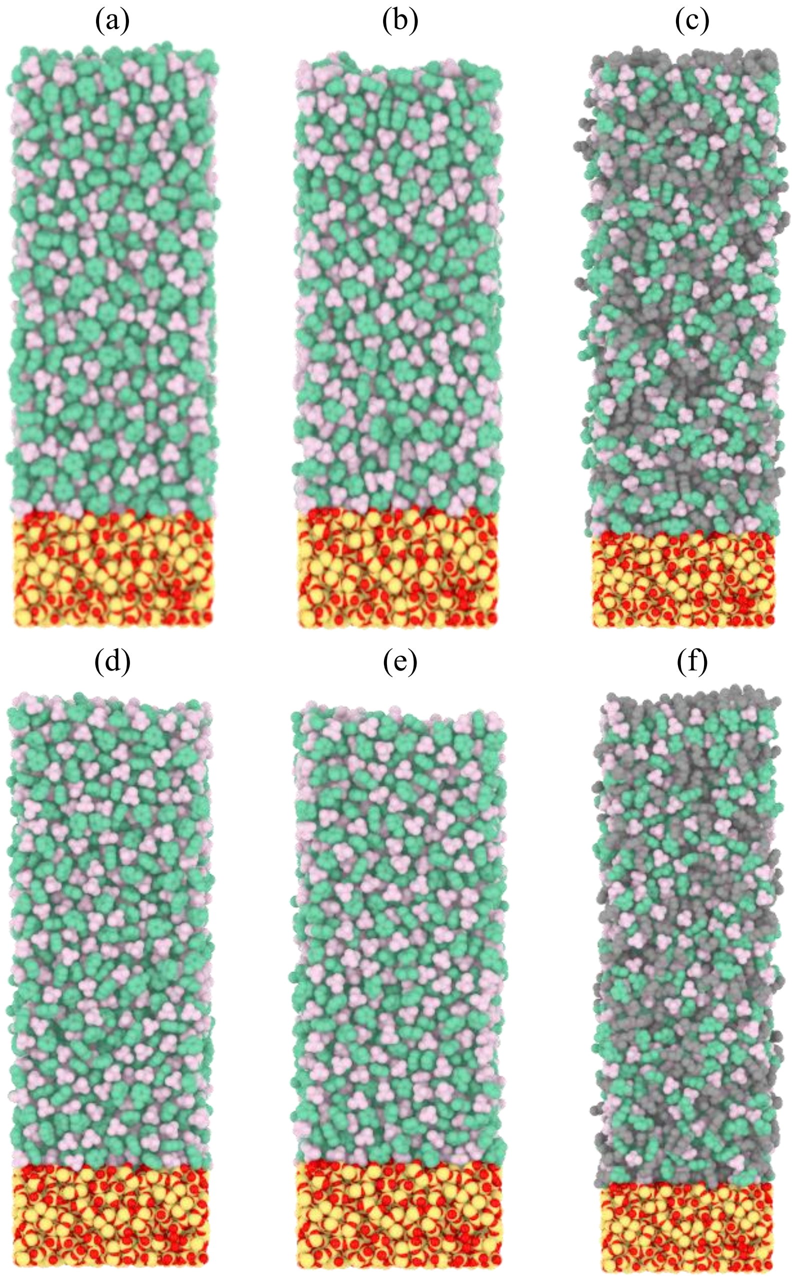

Scheme S1. Snapshots of the MD simulations at 300 K for the IL-glass interface of (a) [4-picH][HSO_4], (b) [MIMH][HSO_4], (c) [C6mim][HSO_4], (d) [4-picH][CH_3SO_3], (e) [MIMH][CH_3SO_3] and (f) [C_6mim][CH_3SO_3]. Green and purple colors represent the polar parts of cations and anions, respectively, and the grey color depicts the nonpolar alkyl tail of the [C_6mim]$^+$ ion. The Si and O atoms of the silica substrate are denoted respectively in yellow and red colors.

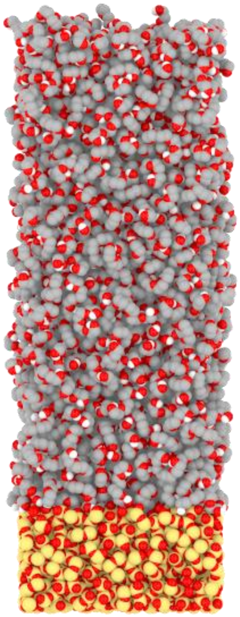

Scheme S2. Snapshot of the MD simulation at 300 K of the PEG200-glass interface. Red color depicts the oxygen atoms, white represents hydrogen atoms of hydroxyl groups, and the grey color depicts carbon atoms in PEG200 moiety. The Si and O atoms of the silica substrate are denoted respectively in yellow and red.

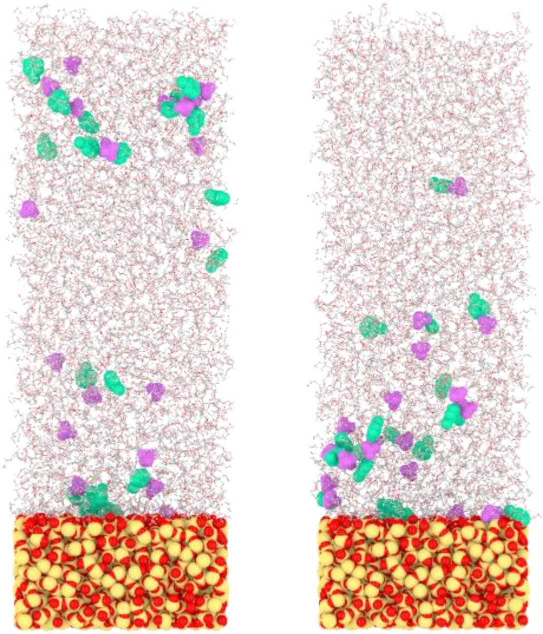

Scheme S3. Snapshots of the MD simulations at 300 K of two independent runs for the mixture PEG200+2% [4-picH][HSO_4] near the glass interface. Green and purple colors represent the polar parts of cations and anions, respectively, while the PEG200 chains are represented as wireframes. The Si and O atoms of the silica substrate are denoted respectively in yellow and red.

Table S1. Force field parameters used in MD simulations of 4-picolinium cation.

[4-picH]$^+$

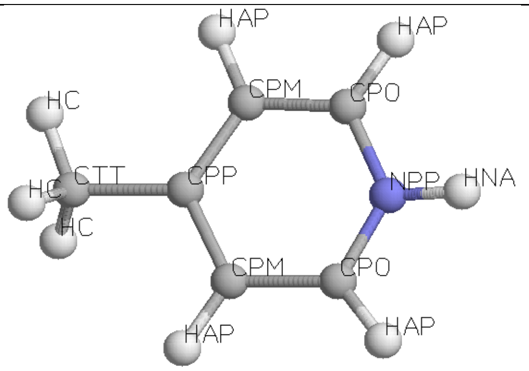

Atom types	Coulomb term q (e)	Lennard-Jones parameters ε (kJ mol^{-1})	σ (Å)
NPP	-0.20	0.71128	3.25
CPO	0.10	0.29288	3.55
CPM	-0.16	0.29288	3.55
CPP	0.28	0.29288	3.55
HNA	0.37	0.00000	0.00
HAP	0.15	0.12552	2.42
CTT	-0.11	0.27614	3.50
HC	0.06	0.12552	2.50
**Bonds**	r_0 **(Å)**	k_r **(kJ mol^{-1} Å^{-2})**	
NPP–HNA	1.010	—	
CPO–HAP	1.080	—	
CPM–HAP	1.080	—	
CTT–HC	1.090	—	
NPP–CPO	1.340	4042.0	
CPO–CPM	1.400	3924.6	
CPM–CPP	1.400	3924.6	
CPP–CTT	1.510	2652.7	
**Angles**	θ_0 **(deg)**	k_θ **(kJ mol^{-1} rad^{-2})**	
CPO–NPP–HNA	118.00	292.90	
CPO–NPP–CPO	120.40	585.80	
CPM–CPO–NPP	120.00	292.90	
NPP–CPO–HAP	120.00	292.90	
CPM–CPO–HAP	120.00	292.90	
CPP–CPM–HAP	120.00	292.90	
CPO–CPM–HAP	120.00	292.90	
CPO–CPM–CPP	120.00	527.20	

CPM–CPP–CPM	120.00	527.20	
CPM–CPP–CTT	120.00	585.80	
CPP–CTT–HC	109.50	418.40	
HC–CT–HC	107.80	276.10	
**Dihedrals**	**V_1 (kJmol^{-1})**	**V_2 (kJ mol^{-1})**	**V_3 (kJmol^{-1})**
CPM–CPO–NPP–HNA	0.0000	30.3340	0.0000
CPO–NPP–CPO–CPM	0.0000	30.3340	0.0000
HAP–CPO–NPP–HNA	0.0000	30.3340	0.0000
CPO–NPP–CPO–HAP	0.0000	30.3340	0.0000
NPP–CPO–CPM–CPP	0.0000	30.3340	0.0000
CPP–CPM–CPO–HAP	0.0000	30.3340	0.0000
NPP–CPO–CPM–HAP	0.0000	30.3340	0.0000
HAP–CPO–CPM–HAP	0.0000	30.3340	0.0000
HAP–CPM–CPP–HAP	0.0000	30.3340	0.0000
CPO–CPM–CPP–CPM	0.0000	30.3340	0.0000
CPM–CPP–CPM–HAP	0.0000	30.3340	0.0000
CTT–CPP–CPM–CPO	0.0000	30.3340	0.0000
CTT–CPP–CPM–HAP	0.0000	30.3340	0.0000
CPM–CPP–CTT–HC	0.0000	0.0000	0.0000
CPO–NPP–CPO–CPM	0.0000	30.3340	0.0000
**Improper dihedrals**	**V_1 (kJmol^{-1})**	**V_2 (kJ mol^{-1})**	**V_3 (kJmol^{-1})**
HNA–NPP–CPO–CPO	0.00000	9.2048	0.0000
HAP–CPO–NPP–CPM	0.00000	9.2048	0.0000
HAP–CPM–CPO–CPP	0.00000	9.2048	0.0000
CTT–CPP–CPM–CPM	0.00000	9.2048	0.0000

Table S2. Force field parameters used in MD simulations of methylimidazolium cation.

[MIMH]$^+$

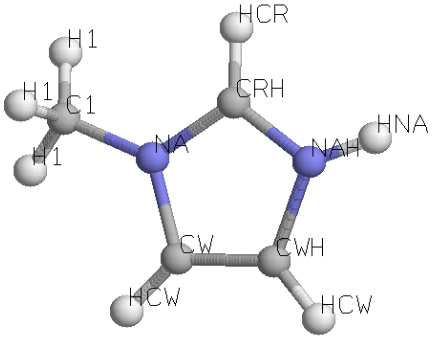

**Atom types**	**Coulomb term** q **(e)**	**Lennard-Jones parameters** ε **(kJ mol^{-1})**	σ **(Å)**
NA	0.15	0.71128	3.25
CRH	0.00	0.29288	3.55
NAH	-0.21	0.71128	3.25
CWH	-0.03	0.29288	3.55
CW	-0.13	0.29288	3.55
C1	-0.17	0.27614	3.50
HCR	0.21	0.12552	2.42
HNA	0.37	0.00000	0.00
HCW	0.21	0.12552	2.42
H1	0.13	0.12552	2.50
**Bonds**	r_0 **(Å)**	k_r **(kJ mol^{-1} Å^{-2})**	
CRH–HCR	1.080	—	
NAH–HNA	1.010	—	
CWH–HCW	1.080	—	
CW–HCW	1.080	—	
C1–H1	1.090	—	
CRH–NAH	1.315	3992.0	
CR–NA	1.315	3992.0	
CWH–NAH	1.378	3574.0	
CW–NA	1.378	3574.0	
CWH–CW	1.341	4352.0	
NA–C1	1.466	2820.0	
**Angles**	θ_0 **(deg)**	k_θ **(kJ mol^{-1} rad^{-2})**	
CRH–NA–C1	126.40	585.80	
CW–NA–CRH	108.00	585.80	
CWH–NAH–CRH	108.00	585.80	

CW–NA–C1	125.60	585.80	
NA–CRH–NAH	109.80	585.80	
NAH–CRH–HCR	125.10	292.90	
NA–CRH–HCR	125.10	292.90	
CRH–NAH–HNA	125.40	292.90	
CWH–NAH–HNA	126.60	292.90	
NAH–CWH–CW	107.10	585.80	
NA–CW–CWH	107.10	585.80	
NAH–CWH–HCW	122.00	292.90	
NA–CW–HCW	122.00	292.90	
CWH–CW–HCW	130.90	292.90	
CW–CWH–HCW	130.90	292.90	
NA–C1–H1	110.70	313.80	
**Dihedrals**	**V_1 (kJ mol^{-1})**	**V_2 (kJ mol^{-1})**	**V_3 (kJ mol^{-1})**
C1–NA–CRH–NAH	0.0000	19.4600	0.0000
CW–NA–CRH–NAH	0.0000	19.4600	0.0000
C1–NA–CRH–HCR	0.0000	19.4600	0.0000
CW–NA–CRH–HCR	0.0000	19.4600	0.0000
CWH–NAH–CRH–NA	0.0000	19.4600	0.0000
CWH–NAH–CRH–HCW	0.0000	19.4600	0.0000
HNA–NAH–CRH–NA	0.0000	19.4600	0.0000
HNA–NAH–CRH–HCR	0.0000	19.4600	0.0000
CRH–NAH–CWH–CW	0.0000	12.5500	0.0000
CRH–NA–CW–CWH	0.0000	12.5500	0.0000
HNA–NAH–CWH–CW	0.0000	12.5500	0.0000
HCW–CW–NA–CRH	0.0000	12.5500	0.0000
HCW–CWH–NAH–CRH	0.0000	12.5500	0.0000
HNA–NAH–CWH–HCW	0.0000	12.5500	0.0000
C1–NA–CW–CWH	0.0000	12.5500	0.0000
C1–NA–CW–HCW	0.0000	12.5500	0.0000
NA–CW–CWH–HCW	0.0000	44.9800	0.0000
NAH–CWH–CW–HCW	0.0000	44.9800	0.0000
HCW–CW–CWH–HCW	0.0000	44.9800	0.0000
NA–CW–CWH–NAH	0.0000	44.9800	0.0000
CRH–NA–C1–H1	0.0000	0.0000	0.0000
CW–NA–C1–H1	0.0000	0.0000	0.5190
**Improper dihedrals**	**V_1 (kJ mol^{-1})**	**V_2 (kJ mol^{-1})**	**V_3 (kJ mol^{-1})**

C1–NA–CW–CRH	0.0000	8.3700	0.0000
HCR–CRH–NAH–NA	0.0000	9.2000	0.0000
HNA–NAH–CWH–CRH	0.0000	8.3700	0.0000
HCW–CW–NA–CWH	0.0000	9.2000	0.0000
HCW–CWH–NAH–CW	0.0000	9.2000	0.0000

Table S3. Force field parameters used in MD simulations of 1-methyl-3-hexylimidazolium cation.

$[C_6mim]^+$

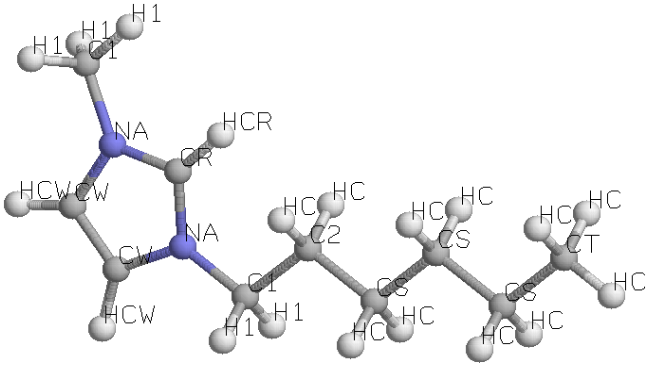

Atom types	Coulomb term<br>q (e)	Lennard-Jones parameters<br>ε (kJ mol^{-1})	 σ (Å)
NA	0.15	0.71128	3.25
CR	-0.11	0.29288	3.55
CW	-0.13	0.29288	3.55
HCR	0.21	0.12552	2.42
HCW	0.21	0.12552	2.42
C1	-0.17	0.27614	3.50
C2	0.01	0.27614	3.50
CS	-0.12	0.27614	3.50
CT	-0.18	0.27614	3.50
H1	0.13	0.12552	2.50
HC	0.06	0.12552	2.50
**Bonds**	**r_0 (Å)**	**k_r (kJ mol^{-1} Å^{-2})**	
CR–HCR	1.080	—	
CW–HCW	1.080	—	
H1–C1	1.090	—	
HC–C2	1.090	—	
HC–CS	1.090	—	
HC–CT	1.090	—	
CR–NA	1.315	3992.0	
CW–NA	1.378	3574.0	
CW-CW	1.341	4352.0	
NA–C1	1.466	2820.0	
C1–C2	1.529	2242.0	
C2–CS	1.529	2242.0	
CS–CS	1.529	2242.0	
CS–CT	1.529	2242.0	

Angles	θ_0 (deg)	k_θ (kJ mol^{-1} rad^{-2})
CR–NA–C1	126.40	585.80
CW–NA–CR	108.00	585.80
CW–NA–C1	125.60	585.80
NA–CR–NA	109.80	585.80
NA–CR–HCR	125.10	292.90
NA–CW–CW	107.10	585.80
NA–CW–HCW	122.00	292.90
CW–CW–HCW	130.90	292.90
NA–C1–H1	110.70	313.80
NA–C1–C2	112.70	488.30
H1–C1–H1	107.80	276.10
HC–C2–HC	107.80	276.10
HC–CS–HC	107.80	276.10
HC–CT–HC	107.80	276.10
C1–C2–HC	110.70	313.80
C2–C1–H1	110.70	313.80
C2–CS–HC	110.70	313.80
CS–C2–HC	110.70	313.80
CS–CT–HC	110.70	313.80
CT–CS–HC	110.70	313.80
C1–C2–CS	112.70	488.30
C2–CS–CS	112.70	488.30
CS–CS–CS	112.70	488.30
CS–CS–CT	112.70	488.30

Dihedrals	V_1 (kJ mol^{-1})	V_2 (kJ mol^{-1})	V_3 (kJ mol^{-1})
C1–NA–CR–NA	0.0000	19.4600	0.0000
CW–NA–CR–NA	0.0000	19.4600	0.0000
C1–NA–CR-HCR	0.0000	19.4600	0.0000
CW–NA–CR-HCR	0.0000	19.4600	0.0000
C1–NA–CR-HCR	0.0000	19.4600	0.0000
CR–NA–CW–CW	0.0000	12.5500	0.0000
C1–NA–CW–CW	0.0000	12.5500	0.0000
CR–NA–CW–HCW	0.0000	12.5500	0.0000
C1–NA–CW–HCW	0.0000	12.5500	0.0000
NA–CW–CW–HCW	0.0000	44.9800	0.0000
HCW–CW–CW–HCW	0.0000	44.9800	0.0000

NA–CW–CW–NA	0.0000	44.9800	0.0000
CR–NA–C1–H1	0.0000	0.0000	0.0000
CW–NA–C1–H1	0.0000	0.0000	0.5190
CR–NA–C1–C2	-5.2691	0.0000	0.0000
CW–NA–C1–C2	-7.1535	6.1064	0.7939
NA–C1–C2–CS	-7.4797	3.1642	-1.2026
NA–C1–C2–HC	0.0000	0.0000	0.3670
C1–C2–CS–HC	0.0000	0.0000	1.2552
CS–C2–C1–H1	0.0000	0.0000	1.2552
C2–CS–CS–HC	0.0000	0.0000	1.2552
CS–CS–C2–HC	0.0000	0.0000	1.2552
CS–CS–CS–HC	0.0000	0.0000	1.2552
CS–CS–CT–HC	0.0000	0.0000	1.2552
CT–CS–CS–HC	0.0000	0.0000	1.2552
H1–C1–C2–HC	0.0000	0.0000	1.2552
HC–C2–CS–HC	0.0000	0.0000	1.2552
HC–CS–CS–HC	0.0000	0.0000	1.2552
HC–CS–CT–HC	0.0000	0.0000	1.2552
C1–C2–CS–CT	5.4392	-0.2092	0.8368
C2–CS–CS–CS	5.4392	-0.2092	0.8368
CS–CS–CS–CS	5.4392	-0.2092	0.8368
CS–CS–CS–CT	5.4392	-0.2092	0.8368
**Improper dihedrals**	**V_1 (kJ mol^{-1})**	**V_2 (kJ mol^{-1})**	**V_3 (kJ mol^{-1})**
CR–CW–NA–C1	0.0000	8.3700	0.0000
NA–NA–CR–HCR	0.0000	9.2000	0.0000
CW–NA–CW-HCW	0.0000	9.2000	0.0000

Table S4. Force field parameters used in MD simulations of hydrogen sulfate anion.

$[HSO_4]^-$

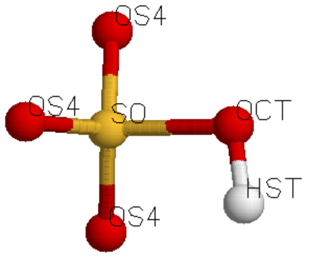

Atom types	Coulomb term q (e)	Lennard-Jones parameters ε (kJ mol^{-1})	σ (Å)
SO	1.18	1.04600	3.55
OS4	-0.65	0.83700	3.15
OCT	-0.60	0.58600	2.90
HST	0.37	0.00000	0.00
**Bonds**	r_0 (Å)	k_r (kJ mol^{-1} Å^{-2})	
HST–OCT	0.950	—	
OS4–SO	1.455	5331.0	
OCT–SO	1.633	1789.6	
**Angles**	θ_0 (deg)	k_θ (kJ mol^{-1} rad^{-2})	
OS4–SO–OS4	114.00	969.00	
OCT–SO–OS4	103.50	1239.60	
HST–OCT–SO	106.40	300.45	
**Dihedrals**	V_1 (kJ mol^{-1})	V_2 (kJ mol^{-1})	V_3 (kJ mol^{-1})
HST–OCT–SO–OS4	0.0000	0.0000	2.4815

Table S5. Force field parameters used in MD simulations of methylsulfonate anion.

$[MeSO_3]^-$

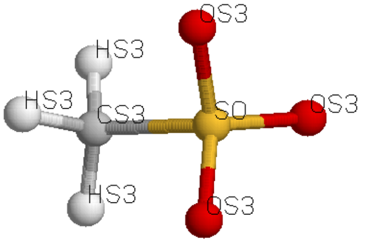

	Coulomb term	Lennard-Jones parameters	
**Atom types**	**q (e)**	**ε (kJ mol^{-1})**	**σ (Å)**
SO	1.18	1.04600	3.55
OS3	-0.68	0.83700	3.15
CS3	-0.14	0.27614	3.50
HS3	0.00	0.12552	2.50
**Bonds**	**r_0 (Å)**	**k_r (kJ mol^{-1} Å^{-2})**	
CS3–HS3	1.090	—	
OS3–SO	1.455	5331.0	
CS3–SO	1.792	1970.0	
**Angles**	**θ_0 (deg)**	**k_θ (kJ mol^{-1} rad^{-2})**	
OS3–SO–OS3	114.00	969.00	
CS3–SO–OS3	114.00	969.00	
CS3–SO–OS3	104.50	870.00	
HS3–CS3–SO	107.30	390.30	
HS3–CS3–HS3	107.80	276.10	
**Dihedrals**	**V_1 (kJ mol^{-1})**	**V_2 (kJ mol^{-1})**	**V_3 (kJ mol^{-1})**
OS3–SO–CS3–HS3	0.0000	0.0000	1.6250

Table S6. Force field parameters used in MD simulations of polyethylene glycol (PEG200).

PEG200

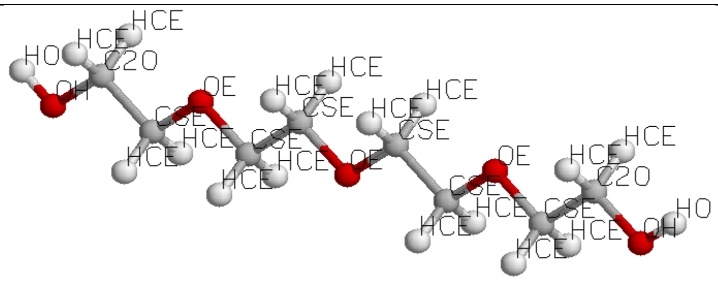

Atom types	Coulomb term<br>q (e)	Lennard-Jones parameters<br>ε (kJ mol^{-1})	 σ (Å)
C2O	0.205	0.27614	3.50
HCE	0.030	0.12552	2.50
OH	-0.683	0.71128	3.12
HO	0.418	0.00000	0.00
CSE	0.140	0.27614	3.50
OE	-0.400	0.58615	2.90
**Bonds**	r_0 **(Å)**	k_r **(kJ mol^{-1} Å^{-2})**	
HCE–C2O	1.090	—	
CSE–HCE	1.090	—	
HO–OH	0.945	—	
C2O–OH	1.410	2677.8	
CSE–C2O	1.529	2242.6	
CSE–OE	1.410	2678.0	
CSE–CSE	1.529	2242.6	
**Angles**	θ_0 **(deg)**	k_θ **(kJ mol^{-1} rad^{-2})**	
HCE–C2O–HCE	107.80	276.10	
HCE–C2O–OH	109.50	292.90	
HCE–C2O–CSE	110.70	313.80	
OH–C2O–CSE	109.50	418.40	
C2O–OH–HO	108.50	460.20	
HCE–CSE–C2O	110.70	313.80	
C2O–CSE–OE	109.50	418.40	
HCE–CSE–HCE	107.80	276.14	
HCE–CSE–OE	109.50	418.40	
CSE–OE–CSE	109.50	502.42	
CSE–CSE–OE	109.50	418.40	
CSE–CSE–HCE	110.70	313.80	
C2O–OH–HO	108.50	460.20	

Dihedrals	V_1 (kJ mol^{-1})	V_2 (kJ mol^{-1})	V_3 (kJ mol^{-1})
HCE–C2O–OH–HO	0.0000	0.0000	1.8828
HO–OH–C2O–CSE	-1.4895	-0.7280	2.0585
HCE–CSE–C2O–HCE	0.0000	0.0000	1.3305
HCE–CSE–C2O–OH	0.0000	0.0000	1.9581
OE–CSE–C2O–HCE	0.0000	0.0000	1.9581
OE–CSE–C2O–OH	-2.3012	0.0000	0.0000
CSE–OE–CSE–C2O	2.7196	-1.0460	2.8033
CSE–OE–CSE–HCE	0.0000	0.0000	3.1798
CSE–CSE–OE–CSE	2.7196	-1.0460	2.8033
HCE–CSE–CSE–OE	0.0000	0.0000	1.9581
HCE–CSE–CSE–HCE	0.0000	0.0000	1.3305
OE–CSE–CSE–OE	-2.3012	0.0000	0.0000
HCE–C2O–OH–HO	0.0000	0.0000	1.8828

Table S6. Force field parameters used in MD simulations of amorphous silica.

**SiO_2**			
	**Coulomb term**	**Lennard-Jones parameters**	
**Atom types**	***q*** **(e)**	ε **(kJ mol^{-1})**	σ **(Å)**
Si4+	2.40	0.32395	4.27
O_2-	-1.20	0.61940	2.78
H_+ (silanol group)	0.41	0.00000	1.00
O (silanol group)	-0.82	0.65314	3.166

Table S7. Final box length (l_b, in nm) and calculated densities (ρ_{MD}, in g.cm^{-3}) for the bulk systems simulated at 300 K.

**System**	l_b	ρ_{MD} / g.cm^{-3}
[4-picH][CH_3SO_3]	5.330	1.2455
[4-picH][HSO_4]	5.126	1.4144
[MIMH][CH_3SO_3]	4.906	1.2529
[MIMH][HSO_4]	4.731	1.4125
[C_6mim][CH_3SO_3]	7.121	1.0857
[C_6mim][HSO_4]	5.521	1.1736
2% [4-picH][HSO_4]	6.693	1.0970
5% [4-picH][HSO_4]	6.740	1.1054

Table S8. Surface area per ion pair (or per PEG200 molecule) at the glass interface (in nm^2) for the different systems studied. The number of ions and molecules were determined by integration of the numerical density profiles between z = 0 nm (glass surface) and z = 0.5 nm (first minima in the density profiles).

compound	100% IL	20% IL	2% IL	0% IL
[4-picH][CH_3SO_3]	0.46			
[4-picH][HSO_4]	0.38	2.4	9.4	
[MIMH][CH_3SO_3]	0.43			
[MIMH][HSO_4]	0.36			
[C_6mim][CH_3SO_3]	0.81			
[C_6mim][HSO_4]	0.72			
PEG200		0.60	0.49	0.46

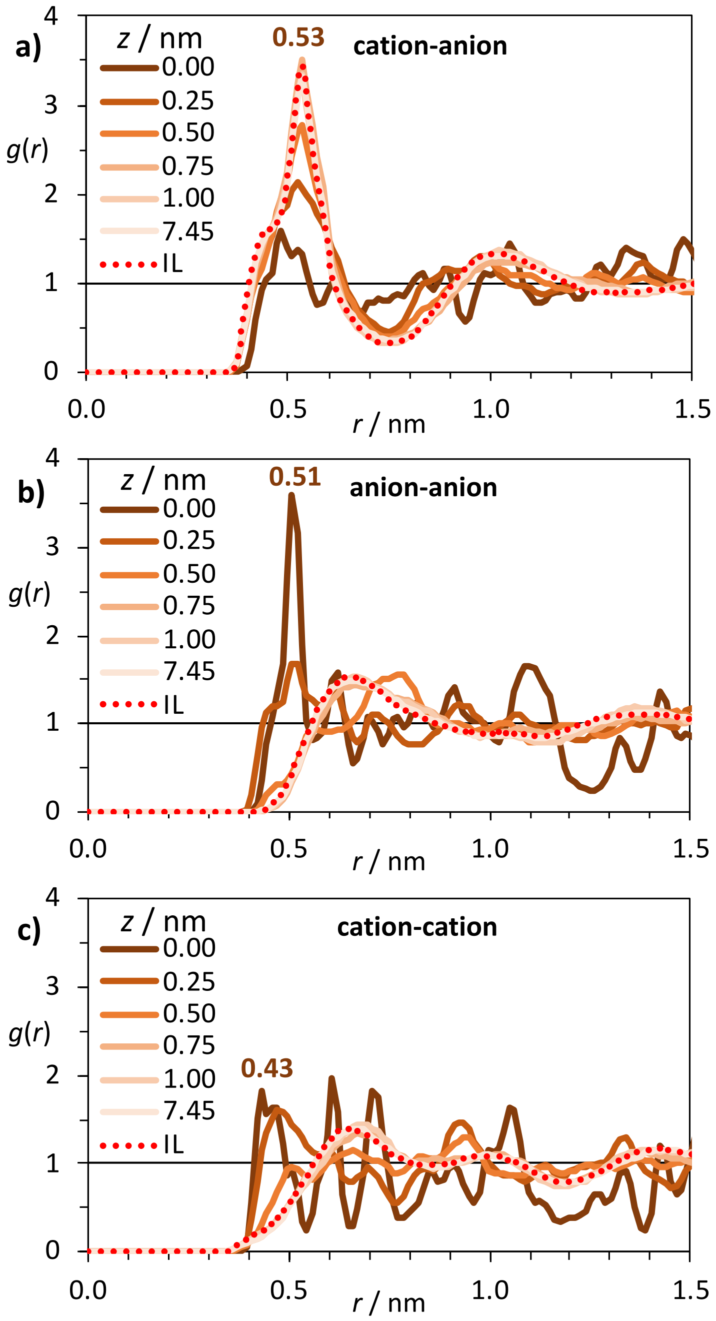

Figure S2. Tangential RDFs between the centers of mass of (a) cations and anions, (b) anions and (c) cations for the [4-picH][CH_3SO_3] protic ionic liquid, collected at several *z* distances (in nm) from the glass interface. The dotted red line represents the corresponding RDF of the isotropic ionic liquid.

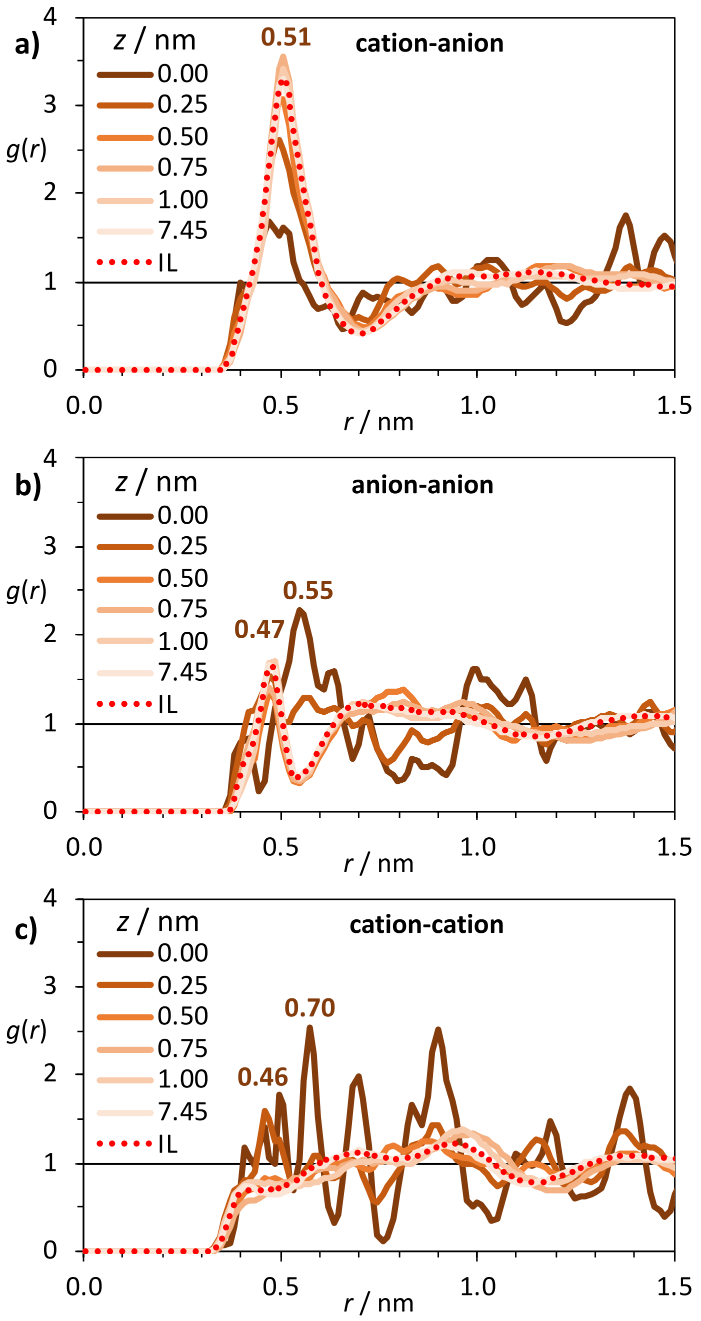

Figure S3. Tangential RDFs between the centers of mass of (a) cations and anions, (b) anions and (c) cations for the [MIMH][HSO_4] protic ionic liquid, collected at several *z* distances (in nm) from the glass interface. The dotted red line represents the corresponding RDF of the isotropic ionic liquid.

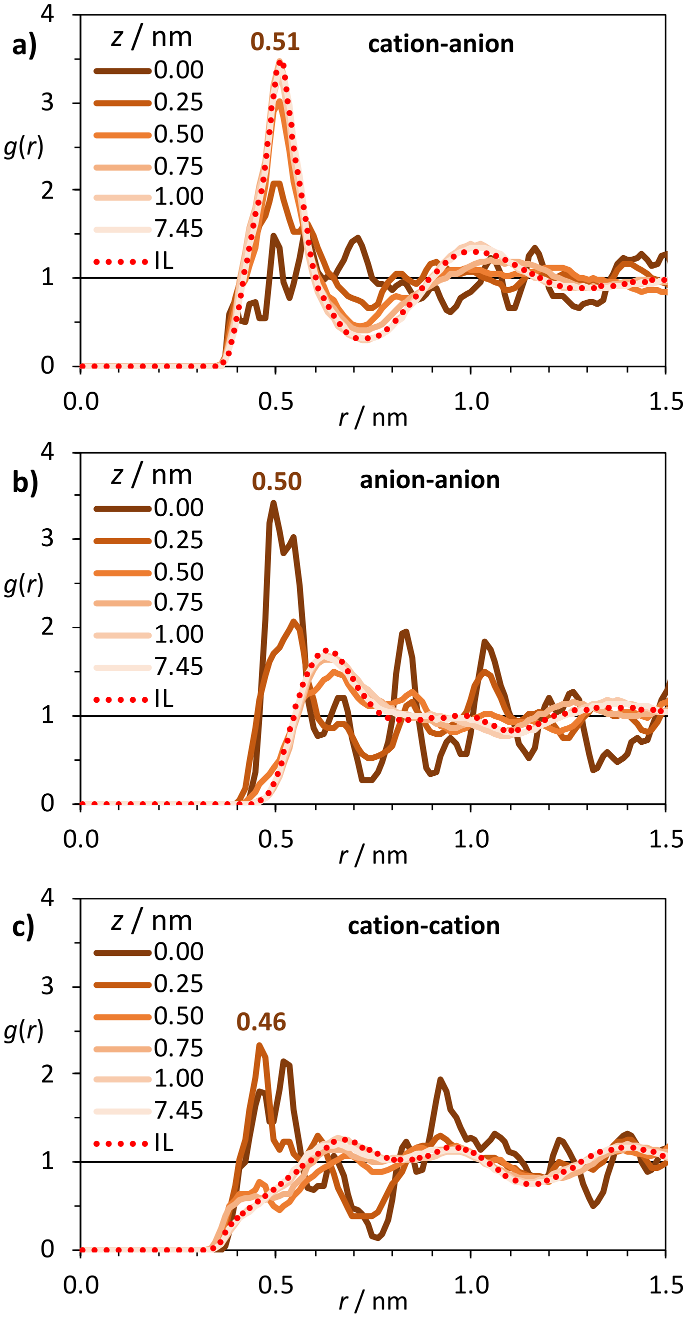

Figure S4. Tangential RDFs between the centers of mass of (a) cations and anions, (b) anions and (c) cations for the [MIMH][CH_3SO_3] protic ionic liquid, collected at several *z* distances (in nm) from the glass interface. The dotted red line represents the corresponding RDF of the isotropic ionic liquid.

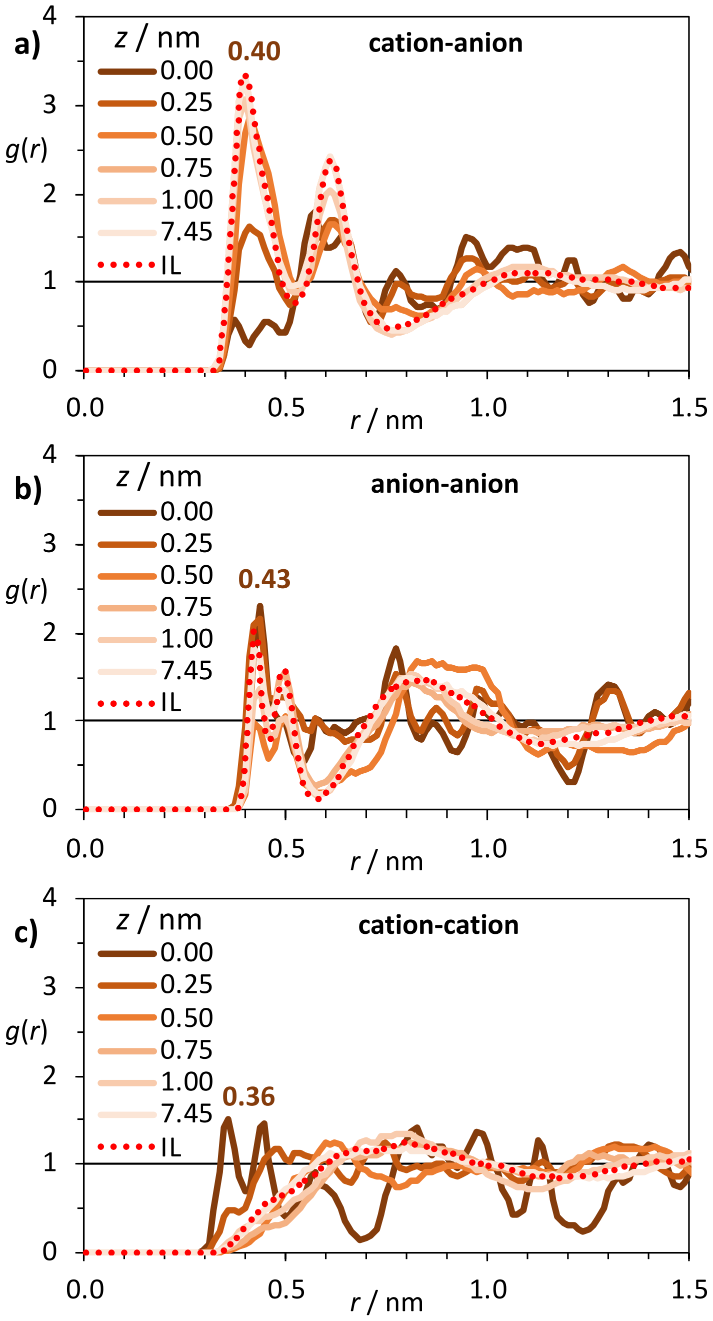

Figure S5. Tangential RDFs between the centers of mass of (a) cations and anions, (b) anions and (c) cations for the [C_6mim][HSO_4] ionic liquid, collected at several *z* distances (in nm) from the glass interface. The dashed line represents the corresponding RDF of the isotropic ionic liquid.

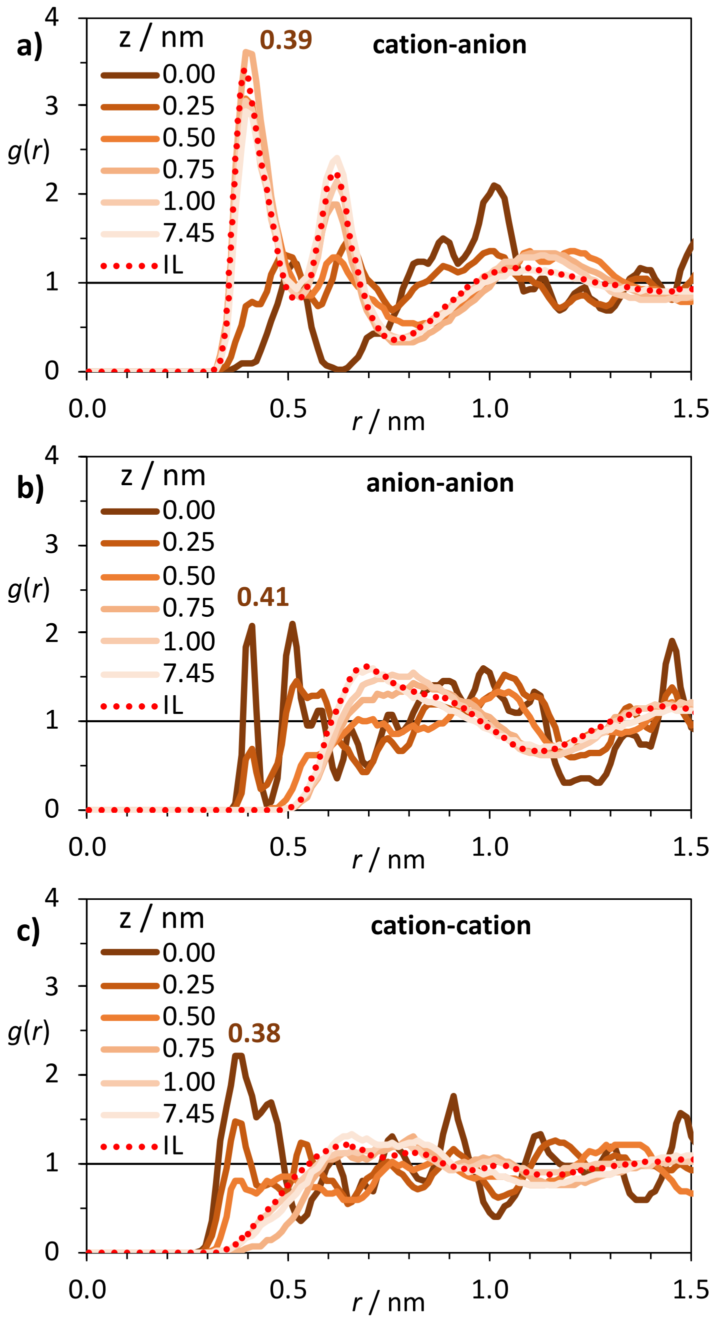

Figure S6. Tangential RDFs between the centers of mass of (a) cations and anions, (b) anions and (c) cations for the [C_6mim][CH_3SO_3] ionic liquid, collected at several *z* distances (in nm) from the glass interface. The dashed line represents the corresponding RDF of the isotropic ionic liquid.

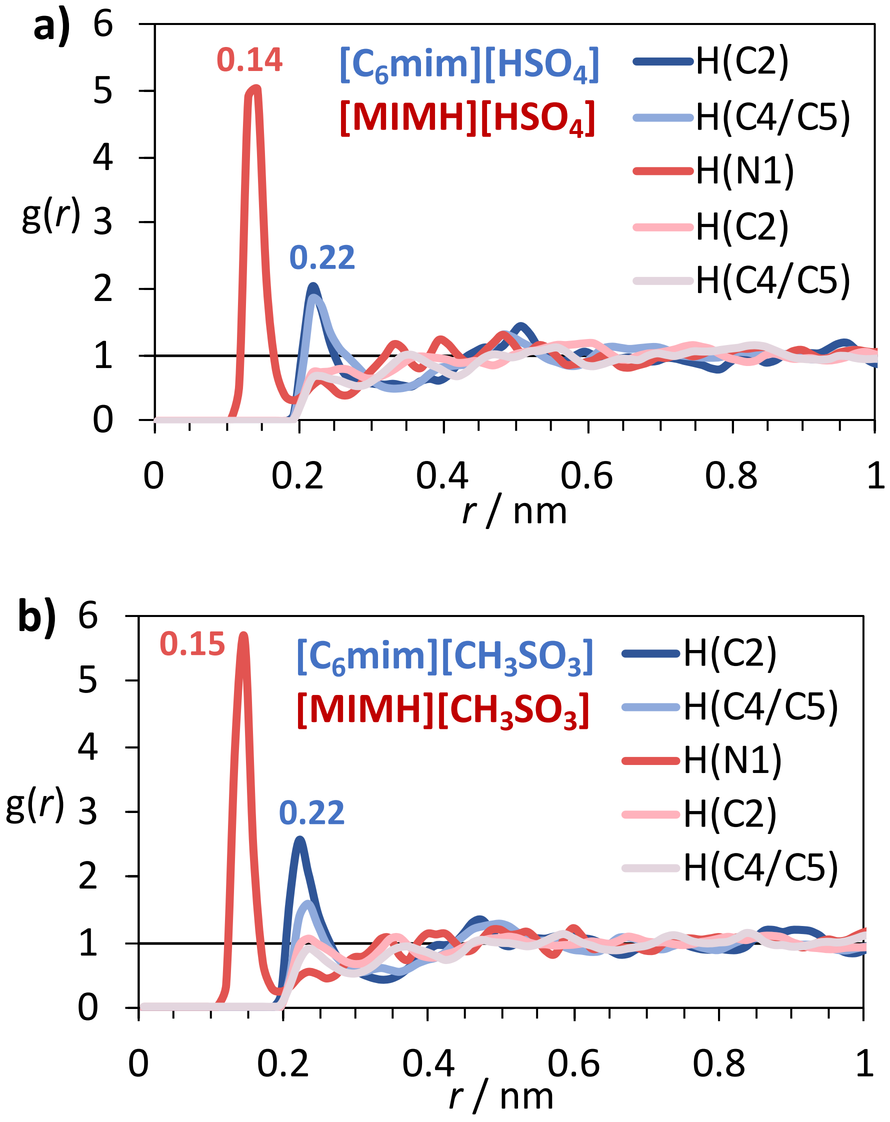

Figure S7. Tangential RDFs between the oxygen atoms of the silica glass (SiO_2) and hydrogen atoms of the aromatic ring of imidazolium-based PILs in (a) [C_6mim][HSO_4] and [MIMH][HSO_4] ILs, and in (b) [C_6mim][CH_3SO_3] and [MIMH][CH_3SO_3] at T = 300 K. The TRDFs were collected for a 0.5 nm-thick layer of IL centered at z = 0 nm. Blue hues denote the H atoms placed at different positions of the [C_6mim]$^+$ ring, while the red tones refer to H atoms of the [MIMH]$^+$ ion.

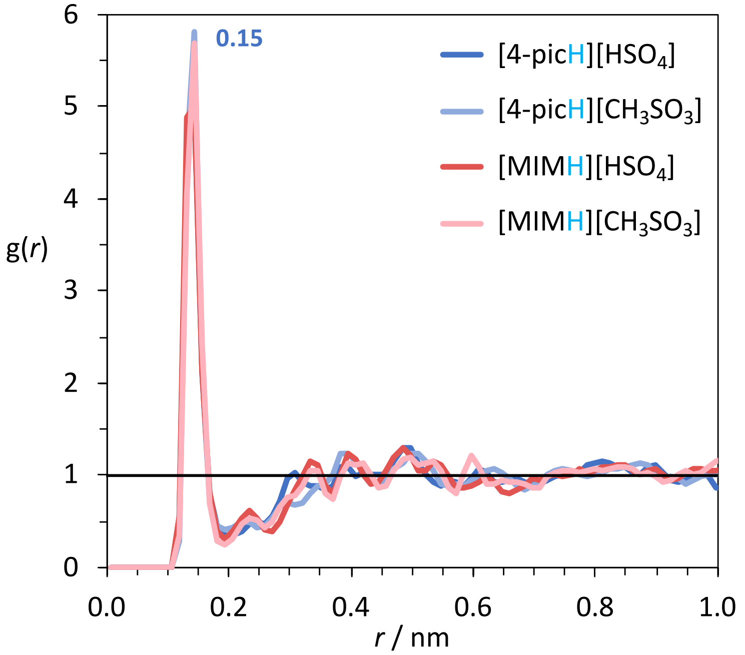

Figure S8. Tangential RDFs between the oxygen atoms of the silica glass (SiO_2) and the most acidic hydrogen atom of the aromatic ring of the cation for [4-picH][HSO_4], [4-picH][CH_3SO_3], [MIMH][HSO_4] and [MIMH][CH_3SO_3] at T = 300 K. The TRDFs were collected for a 0.5 nm-thick layer of IL centered at *z* = 0 nm.

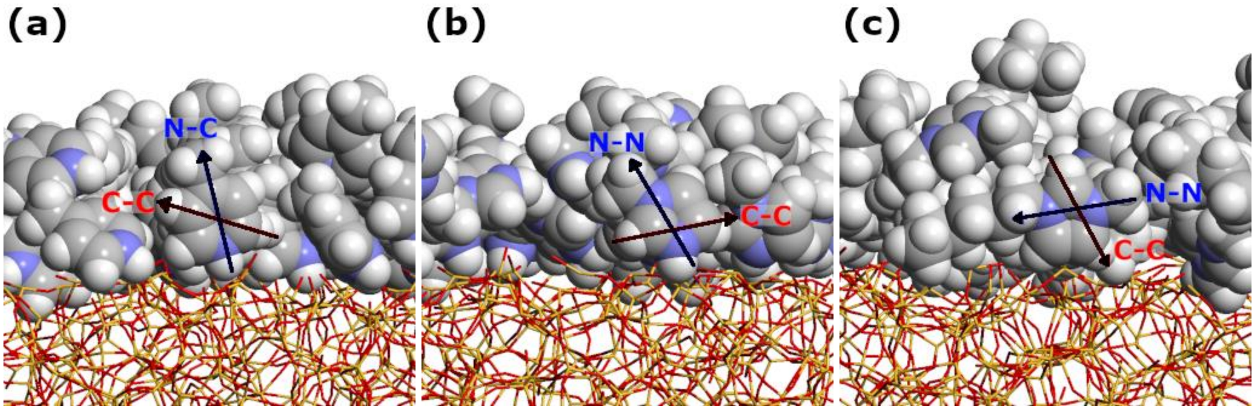

Figure S9. Definition of the direction vectors of (a) [4-picH]$^+$, (b) [MIMH]$^+$ and (c) [C_6mim]$^+$ cations, used in the evaluation of the ring orientational ordering parameter.

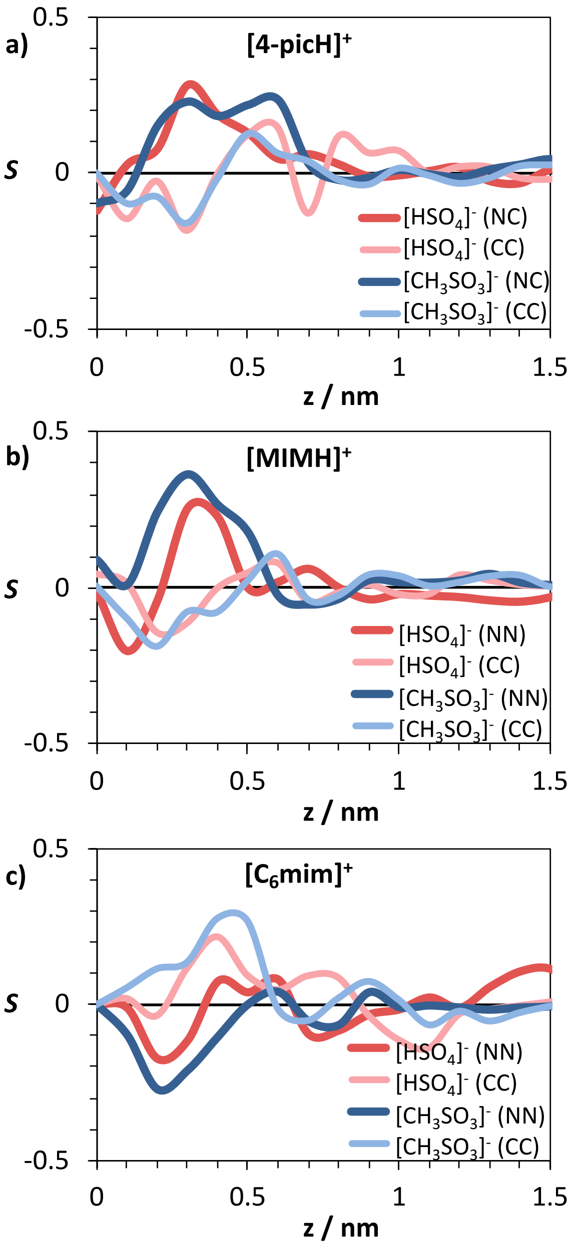

Figure S10. Ring orientational ordering parameter S as a function of the distance to the glass surface for the ILs studied. The reference axis is the surface normal. The NN, NC and CC denote direction vectors of the cations defined in Figure S9. The angles 0º (S = 1) and 90º (S = 0.5) represent perfectly aligned and perfectly perpendicular axis with respect to the surface normal, respectively, while the value $S = 0$ corresponds to an isotropic distribution or a system perfectly oriented at the magic angle 54.7º.

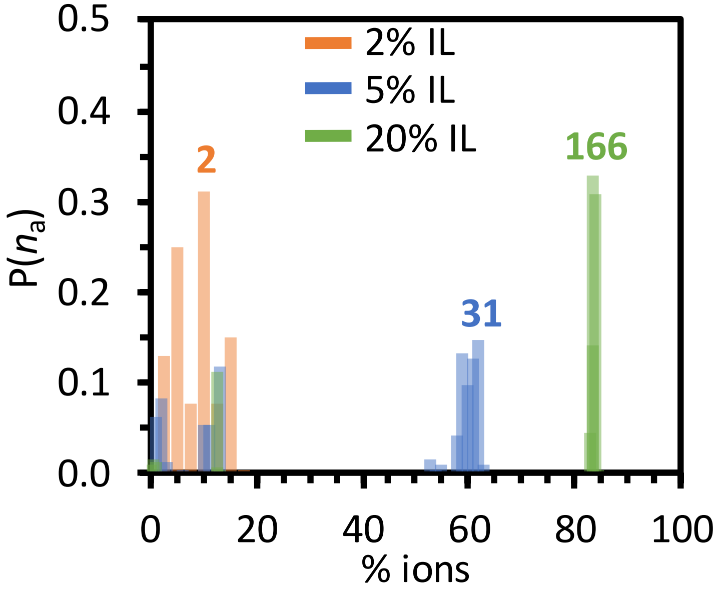

Figure S11. Distribution function of cation-anion aggregates $P(n_a)$ in PEG200+[4-picH][HSO_4] systems as a function of the total % of ions. The numbers on top of the columns represent the ion pairs found for the aggregate population with the highest probability.

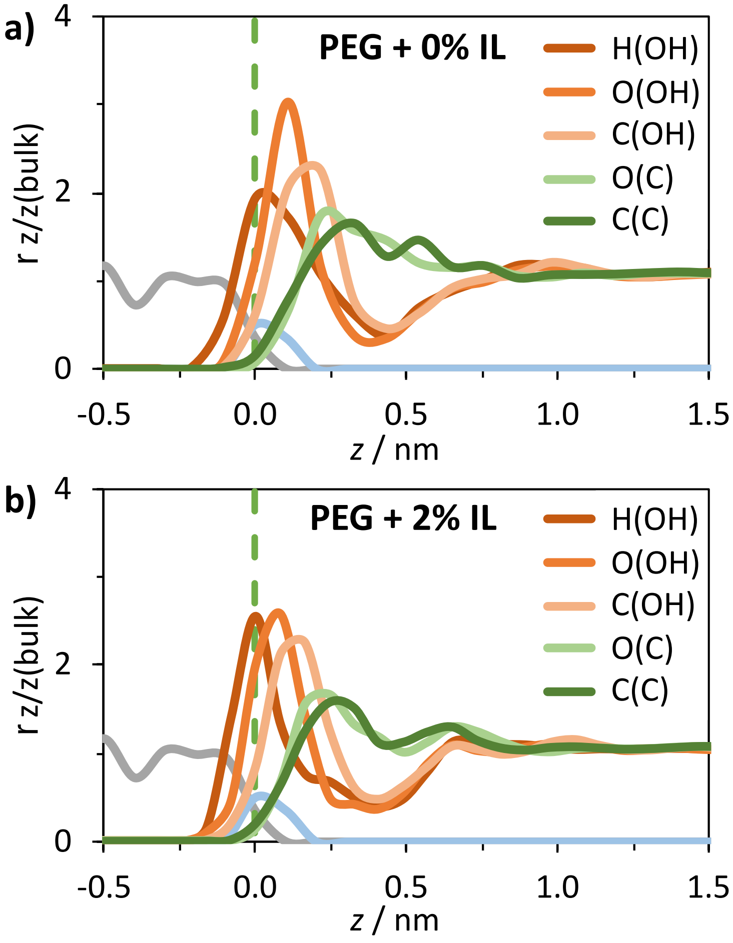

Figure S12. Number density profiles along the direction normal to glass surface for OH groups and CH_2CH_2O units of (a) PEG200 and (b) PEG200+2% [4-picH][HSO_4] (values normalized to nominal values in case of a homogeneous isotropic bulk). The atom types H(OH) and O(OH) refer to hydroxyl atoms, C(OH) is the carbon atom bonded to the hydroxyl group, O(C) and C(C) designate atoms of the alkoxy group. The vertical dashed line represents z = 0 nm and is defined as the outermost atoms at the glass surface (O atoms of silanol groups, represented in light blue). The grey line denotes the Si atoms in the solid substrate.

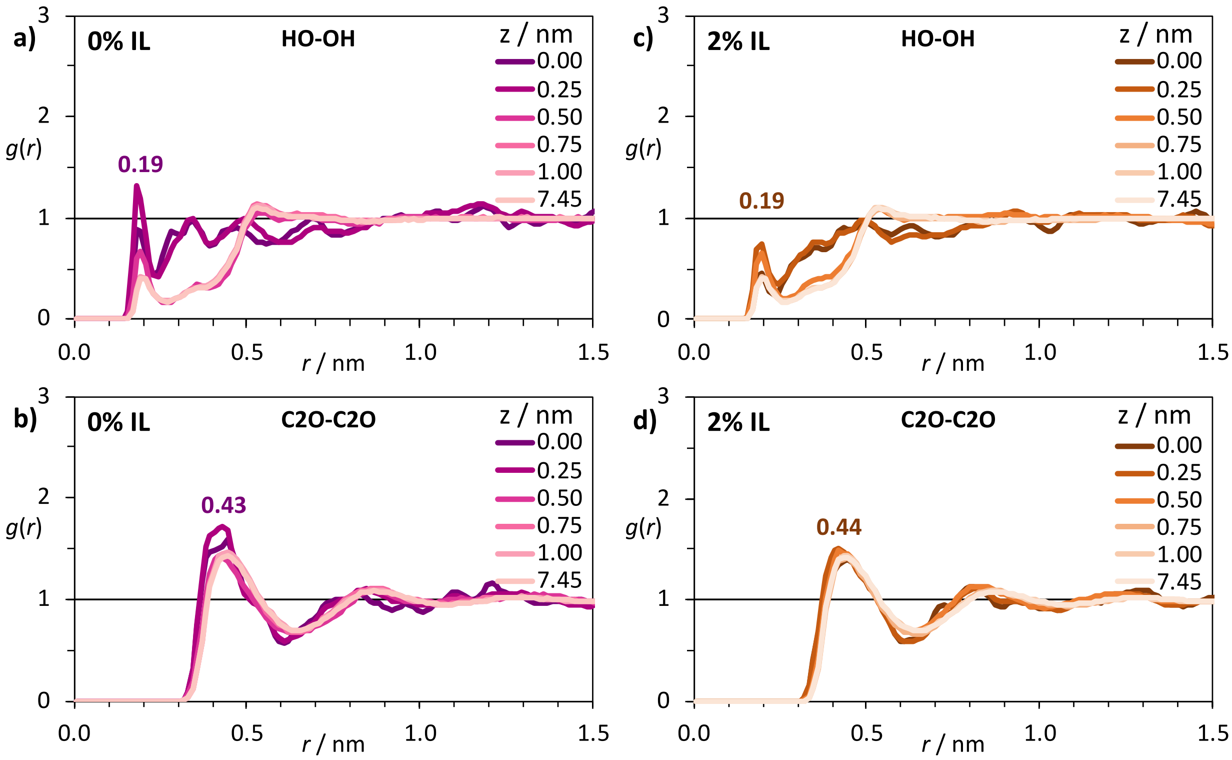

Figure S13. Tangential RDFs of selected centers for pure PEG200 (a and b) and PEG200+2% [4-picH][HSO_4] (c and d), collected at several *z* distances (in nm) from the glass interface. HO and OH represent respectively hydrogen and oxygen atoms of the hydroxyl groups of PEG200, while C2O depicts the carbon atom bonded to the hydroxyl group.

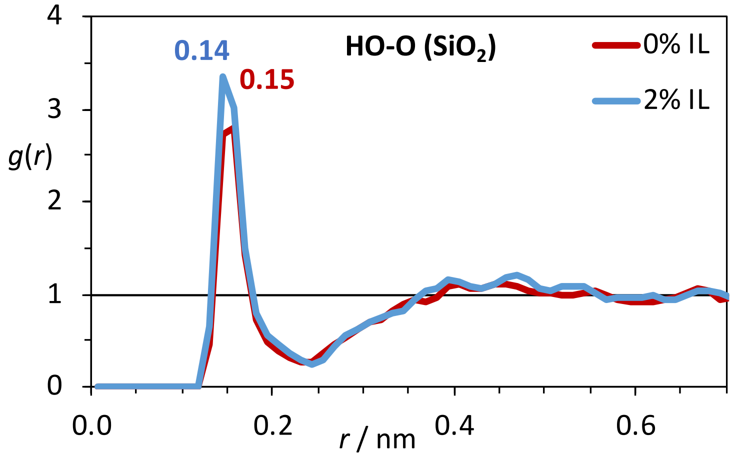

Figure S14. Tangential RDFs between the oxygen atoms of the silica glass (SiO_2) and hydrogen atoms of hydroxyl groups of PEG200 in (a) PEG200 and (b) PEG200+2% [4-picH][HSO_4]. The TRDFs were collected for a 0.5 nm-thick layer of IL centered at z = 0 nm.

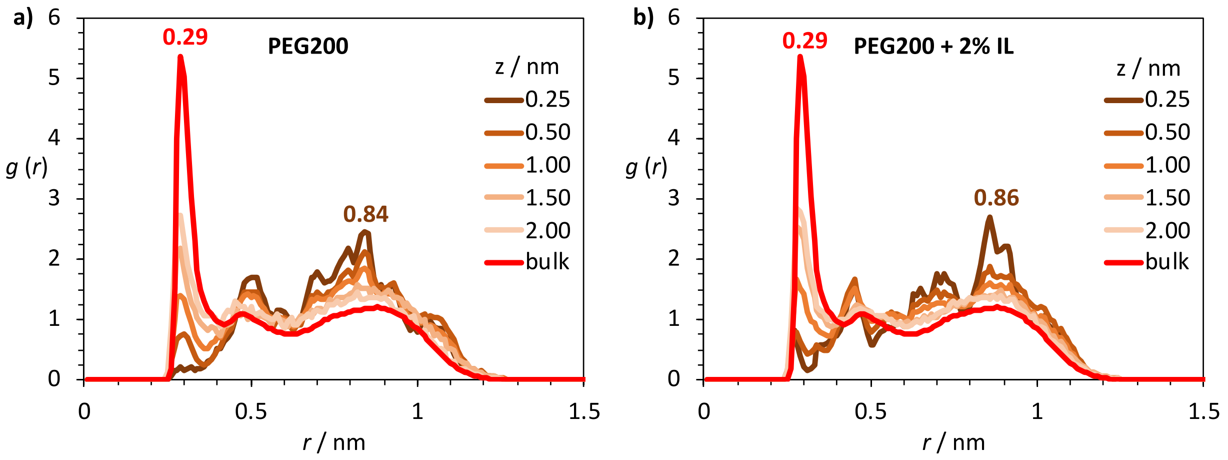

Figure S15. Intramolecular distance distribution function of the OH groups in PEG200 chain as a function of the distance to the glass surface (in nm) for (a) pure PEG200 and (b) PEG200 + 2% [4-picH][HSO_4].

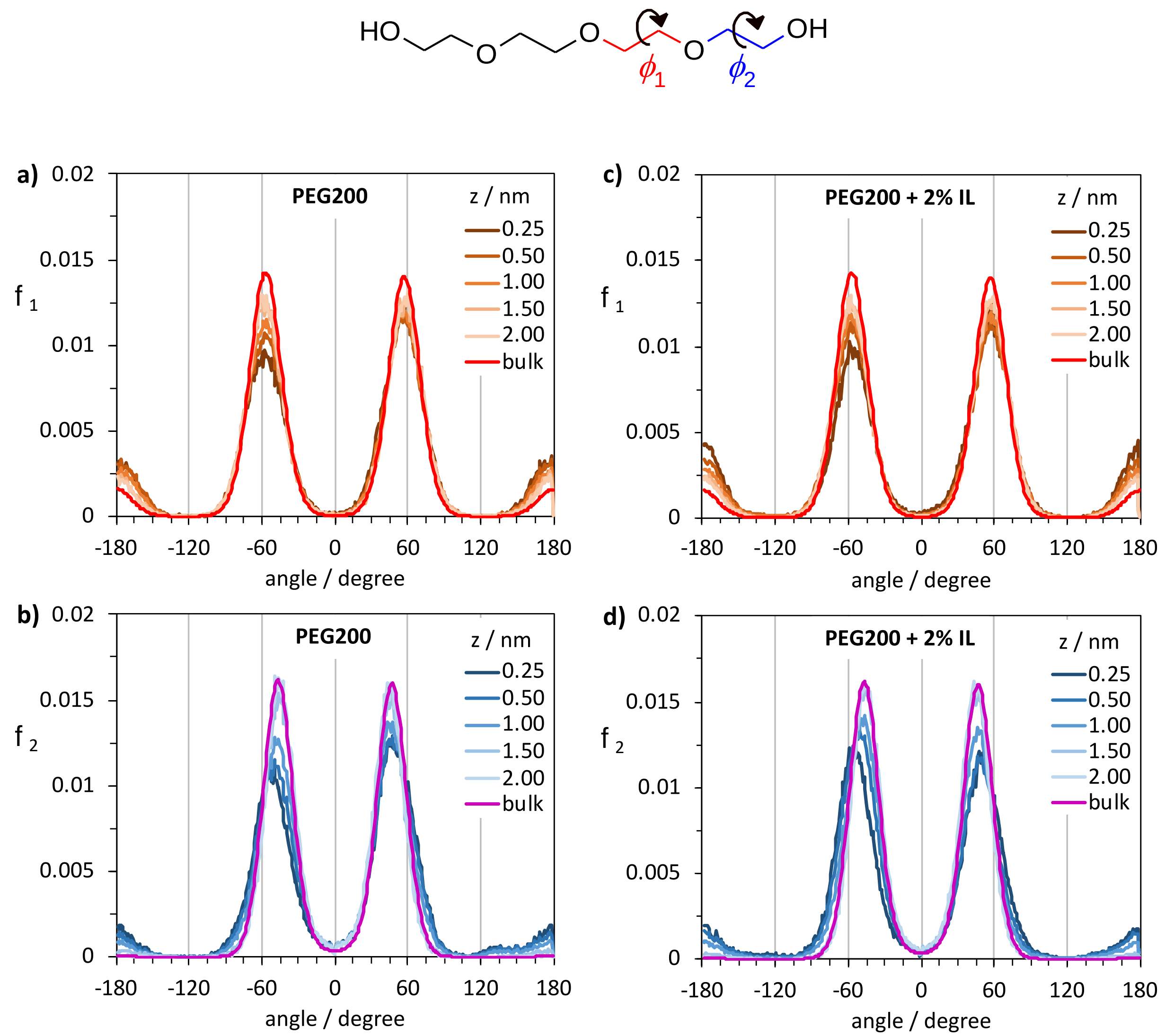

Figure S16. Torsion angle distributions of PEG200 chains as a function of the distance to the glass surface (in nm) for (a and b) pure PEG200 and (c and d) PEG200 + 2% [4-picH][HSO_4]. The ϕ_1 represents the dihedral angle -O(R)-C-C-O(R)-, while ϕ_2 denotes the torsion angle -O(R)-C-C-O(H).
